# The Concise Guide to PHARMACOLOGY 2015/16: Voltage‐gated ion channels

**DOI:** 10.1111/bph.13349

**Published:** 2015-12-09

**Authors:** Stephen PH Alexander, William A Catterall, Eamonn Kelly, Neil Marrion, John A Peters, Helen E Benson, Elena Faccenda, Adam J Pawson, Joanna L Sharman, Christopher Southan, Jamie A Davies, CGTP Collaborators

**Affiliations:** ^1^ School of Biomedical Sciences University of Nottingham Medical School Nottingham NG7 2UH UK; ^2^ Department of Pharmacology University of Washington Seattle 98195‐7280 WA USA; ^3^ School of Physiology and Pharmacology University of Bristol Bristol BS8 1TD UK; ^4^ Neuroscience Division, Medical Education Institute, Ninewells Hospital and Medical School University of Dundee Dundee DD1 9SY UK; ^5^ Centre for Integrative Physiology University of Edinburgh Edinburgh EH8 9XD UK

## Abstract

The Concise Guide to PHARMACOLOGY 2015/16 provides concise overviews of the key properties of over 1750 human drug targets with their pharmacology, plus links to an open access knowledgebase of drug targets and their ligands (www.guidetopharmacology.org), which provides more detailed views of target and ligand properties. The full contents can be found at http://onlinelibrary.wiley.com/doi/10.1111/bph.13350/full. Voltage‐gated ion channels are one of the eight major pharmacological targets into which the Guide is divided, with the others being: G protein‐coupled receptors, ligand‐gated ion channels, other ion channels, nuclear hormone receptors, catalytic receptors, enzymes and transporters. These are presented with nomenclature guidance and summary information on the best available pharmacological tools, alongside key references and suggestions for further reading. The Concise Guide is published in landscape format in order to facilitate comparison of related targets. It is a condensed version of material contemporary to late 2015, which is presented in greater detail and constantly updated on the website www.guidetopharmacology.org, superseding data presented in the previous Guides to Receptors & Channels and the Concise Guide to PHARMACOLOGY 2013/14. It is produced in conjunction with NC‐IUPHAR and provides the official IUPHAR classification and nomenclature for human drug targets, where appropriate. It consolidates information previously curated and displayed separately in IUPHAR‐DB and GRAC and provides a permanent, citable, point‐in‐time record that will survive database updates.

## Conflict of interest

The authors state that there are no conflicts of interest to declare.

## Family structure

5905 CatSper and Two‐Pore channels

5907 Cyclic nucleotide‐regulated channels

5909 Potassium channels

5910 Calcium‐activated potassium channels

5912 Inwardly rectifying potassium channels

5915 Two‐P potassium channels

5917 Voltage‐gated potassium channels

5920 Transient Receptor Potential channels

5934 Voltage‐gated calcium channels

5936 Voltage‐gated proton channel

5937 Voltage‐gated sodium channels

## CatSper and Two‐Pore channels

### Overview

CatSper channels (CatSper1‐4, **nomenclature as agreed by NC‐IUPHAR**[**64**]) are putative 6TM, voltage‐gated, calcium permeant channels that are presumed to assemble as a tetramer of *α*‐like subunits and mediate the current I_*CatSper*_[171]. In mammals, CatSper subunits are structurally most closely related to individual domains of voltage‐activated calcium channels (Ca_*v*_) [308]. CatSper1 [308], CatSper2 [302] and CatSpers 3 and 4 [155, 221, 299], in common with a putative 2TM auxiliary CatSper*β* protein [218] and two putative 1TM associated CatSper*γ* and CatSper*δ* proteins [59, 382], are restricted to the testis and localised to the principle piece of sperm tail.

Two‐pore channels (TPCs) are structurally related to CatSpers, Ca_*V*_s and Na_*V*_s. TPCs have a 2x6TM structure with twice the number of TMs of CatSpers and half that of Ca_*V*_s. There are three animal TPCs (TPC1‐TPC3). Humans have TPC1 and TPC2, but not TPC3. TPC1 and TPC2 are localized in endosomes and lysosomes [39]. TPC3 is also found on the plasma membrane and forms a voltage‐activated, non‐inactivating Na^+^ channel [40]. All the three TPCs are Na^+^‐selective under whole‐cell or whole‐organelle patch clamp recording [41, 42, 404]. The channels may also conduct Ca^2+^[243].

**Table:** 

Nomenclature	CatSper1
HGNC, UniProt	*CATSPER1*, Q8NEC5
Activators	CatSper1 is constitutively active, weakly facilitated by membrane depolarisation, strongly augmented by intracellular alkalinisation. In human, but not mouse, spermatozoa progesterone (EC_50_ 8 nM) also potentiates the CatSper current (I_*CatSper*_). [215, 343]
Functional Characteristics	Calcium selective ion channel (Ba^2+^>Ca^2+^≫Mg^2+^≫Na^+^); quasilinear monovalent cation current in the absence of extracellular divalent cations; alkalinization shifts the voltage‐dependence of activation towards negative potentials [V_1/2_ @ pH 6.0 = +87 mV (mouse); V_1/2_ @ pH 7.5 = +11mV (mouse) or pH 7.4 = +85 mV (human)]; required for I_*CatSper*_ and male fertility (mouse and human)
Channel blockers	ruthenium red (Inhibition) (pIC_50_ 5) [171] – Mouse, HC‐056456 (pIC_50_ 4.7) [46], Cd^2+^ (Inhibition) (pIC_50_ 3.7) [171] – Mouse, Ni^2+^ (Inhibition) (pIC_50_ 3.5) [171] – Mouse
Selective channel blockers	NNC55‐0396 (Inhibition) (pIC_50_ 5.7) [‐80mV – 80mV] [215, 343], mibefradil (Inhibition) (pIC_50_ 4.4–4.5) [343]

**Table:** 

Nomenclature	CatSper2	CatSper3	CatSper4
HGNC, UniProt	*CATSPER2*, Q96P56	*CATSPER3*, Q86XQ3	*CATSPER4*, Q7RTX7
Functional Characteristics	Required for I_*CatSper*_ and male fertility(mouse and human)	Required for I_*CatSper*_ and male fertility (mouse)	Required for I_*CatSper*_ and male fertility (mouse)

**Table:** 

Nomenclature	TPC1	TPC2
HGNC, UniProt	*TPCN1*, Q9ULQ1	*TPCN2*, Q8NHX9
Functional Characteristics	Organelle voltage‐gated Na^+^‐selective channel (Na^+^≫K^+^≫Ca^2+^); Required for the generation of action potential‐like long depolarization in lysosomes. Voltage‐dependence of activation is sensitive to luminal pH (determined from lysosomal recordings). *ψ*_1/2_ @ pH4.6 = +91 mV; *ψ*_1/2_ @ pH6.5 = +2.6 mV. Maximum activity requires PI(3,5)P_2_ and reduced [ATP]	Organelle voltage‐independent Na^+^‐selective channel (Na^+^≫K^+^≫Ca^2+^). Sensitive to the levels of PI(3,5)P_2_. Activated by decreases in [ATP] or depletion of extracellular amino acids
Activators	phosphatidyl (3,5) inositol bisphosphate (pEC_50_ 6.5) [41]	phosphatidyl (3,5) inositol bisphosphate (pEC_50_ 6.4) [387]
Channel blockers	verapamil (Inhibition) (pIC_50_ 4.6) [41], Cd^2+^ (Inhibition) (pIC_50_ 3.7) [41]	verapamil (Inhibition) (pIC_50_ 5) [387]

### Comments

CatSper channel subunits expressed singly, or in combination, fail to functionally express in heterologous expression systems [302, 308]. The properties of CatSper1 tabulated above are derived from whole cell voltage‐clamp recordings comparing currents endogenous to spermatozoa isolated from the *corpus epididymis* of wild‐type and*Catsper1* ^(−/−)^ mice [171] and also mature human sperm [215, 343]. I_*CatSper*_ is also undetectable in the spermatozoa of *Catsper2* ^(−/−)^,*Catsper3* ^(−/−)^, *Catsper4* ^(−/−)^, or CatSper*δ* ^(−/−)^ mice, and CatSper 1 associates with CatSper 2, 3, 4, *β*, *γ*, and *δ*[59, 218, 299]. Moreover, targeted disruption of *Catsper1, 2, 3,* *4*, or *δ* genes results in an identical phenotype in which spermatozoa fail to exhibit the hyperactive movement (whip‐like flagellar beats) necessary for penetration of the egg *cumulus* and *zona pellucida* and subsequent fertilization. Such disruptions are associated with a deficit in alkalinization and depolarization‐evoked Ca^2+^ entry into spermatozoa [47, 59, 299]. Thus, it is likely that the CatSper pore is formed by a heterotetramer of CatSpers1‐4 [299] in association with the auxiliary subunits (*β*, *γ*, *δ*) that are also essential for function [59]. CatSper channels are required for the increase in intracellular Ca^2+^ concentration in sperm evoked by egg *zona pellucida* glycoproteins [404]. Mouse and human sperm swim against the fluid flow and Ca^2+^ signaling through CatSper is required for the rheotaxis [239]. *In vivo*, CatSper1‐null spermatozoa cannot ascend the female reproductive tracts efficiently [60, 135]. It has been shown that CatSper channels form four linear Ca^2+^ signaling domains along the flagella, which orchestrate capacitation‐associated tyrosine phosphorylation [60].The driving force for Ca^2+^ entry is principally determined by a mildly outwardly rectifying K^+^ channel (KSper) that, like CatSpers, is activated by intracellular alkalinization [253]. Mouse KSper is encoded by *mSlo3*, a protein detected only in testis [235, 253, 419]. In human sperm, such alkalinization may result from the activation of H_*v*_1, a proton channel [216].

Mutations in CatSpers are associated with syndromic and non‐syndromic male infertility [128]. In human ejaculated spermatozoa, progesterone (<50 nM) potentiates the CatSper current by a non‐genomic mechanism and acts synergistically with intracellular alkalinisation [215, 343]. Sperm cells from infertile patients with a deletion in CatSper2 gene lack I_*CatSper*_ and the progesterone response [331]. In addition, certain prostaglandins (*e.g.* PGF_1*α*_, PGE_1_) also potentiate CatSper mediated currents [215, 343].

In human sperm, CatSper channels are also activated by various small molecules including endocrine disrupting chemicals (EDC) and proposed as a polymodal sensor [35, 35].

TPCs are the major Na^+^ conductance in lysosomes; knocking out TPC1 and TPC2 eliminates the Na^+^ conductance and renders the organelle's membrane potential insensitive to changes in [Na^+^] (31). The channels are regulated by luminal pH [41], PI(3,5)P_2_[387], intracellular ATP and extracellular amino acids [42]. TPCs are also involved in the NAADP‐activated Ca^2+^ release from lysosomal Ca^2+^ stores [39, 243]. Mice lacking TPCs are viable but have phenotypes including compromised lysosomal pH stability, reduced physical endurance [42], resistance to Ebola viral infection [314] and fatty liver [110]. No major human disease‐associated TPC mutation has been reported.

### Further Reading

Calcraft PJ *et al*. (2009) NAADP mobilizes calcium from acidic organelles through two‐pore channels. *Nature* **459**: 596‐600 [PMID:19387438]

Cang C *et al*. (2014) The voltage‐gated sodium channel TPC1 confers endolysosomal excitability. *Nat. Chem. Biol.* **10**: 463‐9 [PMID:24776928]

Cang C *et al*. (2013) mTOR regulates lysosomal ATP‐sensitive two‐pore Na(+) channels to adapt to metabolic state. *Cell* **152**: 778‐90 [PMID:23394946]

Clapham DE *et al*. (2005) International Union of Pharmacology. L. Nomenclature and structure‐function relationships of CatSper and two‐pore channels. *Pharmacol. Rev.* **57**: 451‐4 [PMID:16382101]

Hildebrand MS *et al*. (2010) Genetic male infertility and mutation of CATSPER ion channels. *Eur. J. Hum. Genet.* **18**: 1178‐84 [PMID:20648059]

Kirichok Y *et al*. (2011) Rediscovering sperm ion channels with the patch‐clamp technique. *Mol. Hum. Reprod.* **17**: 478‐99 [PMID:21642646]

Lishko PV *et al*. (2010) The role of Hv1 and CatSper channels in sperm activation. *J. Physiol. (Lond.)* **588**: 4667‐72 [PMID:20679352]

Ren D *et al*. (2010) Calcium signaling through CatSper channels in mammalian fertilization. *Physiology (Bethesda)* **25**: 165‐75 [PMID:20551230]

Wang X *et al*. (2012) TPC proteins are phosphoinositide‐ activated sodium‐selective ion channels in endosomes and lysosomes. *Cell* **151**: 372‐83 [PMID:23063126]

## Cyclic nucleotide‐regulated channels

### Overview

Cyclic nucleotide‐gated (CNG) channels are responsible for signalling in the primary sensory cells of the vertebrate visual and olfactory systems. **A standardised nomenclature for CNG channels has been proposed by the NC‐IUPHAR subcommittee on voltage‐gated ion channels** [**138**].

CNG channels are voltage‐independent cation channels formed as tetramers. Each subunit has 6TM, with the pore‐forming domain between TM5 and TM6. CNG channels were first found in rod photoreceptors [96, 166], where light signals through rhodopsin and transducin to stimulate phosphodiesterase and reduce intracellular cyclic GMP level. This results in a closure of CNG channels and a reduced ‘dark current’. Similar channels were found in the cilia of olfactory neurons [252] and the pineal gland [86]. The cyclic nucleotides bind to a domain in the C terminus of the subunit protein: other channels directly binding cyclic nucleotides include HCN, eag and certain plant potassium channels.

**Table:** 

Nomenclature	CNGA1	CNGA2	CNGA3	CNGB3
HGNC, UniProt	*CNGA1*, P29973	*CNGA2*, Q16280	*CNGA3*, Q16281	*CNGB3*, Q9NQW8
Activators	cyclic GMP (EC_50_ 30 *μ*M) ≫cyclic AMP	cyclic GMP cyclic AMP (EC_50_ 1 *μ*M)	cyclic GMP (EC_50_ 30 *μ*M) ≫cyclic AMP	–
Functional Characteristics	*γ* = 25‐30 pS *P*_*Ca*_/*P*_*Na*_ = 3.1	*γ* = 35 pS *P*_*Ca*_/*P*_*Na*_ = 6.8	*γ* = 40 pS *P*_*Ca*_/*P*_*Na*_ = 10.9	–
Inhibitors	–	–	L‐(cis)‐diltiazem	–
Channel blockers	dequalinium (Antagonist) (pIC_50_ 6.7) [0mV] [312], L‐(cis)‐diltiazem (Antagonist) (p*K*_*i*_ 4) [‐80mV – 80mV] [53]	dequalinium (Antagonist) (pIC_50_ 5.6) [0mV] [311]	–	L‐(cis)‐diltiazem (Antagonist) (pIC_50_ 5.5) [0mV] [102] – Mouse

### Comments

CNGA1, CNGA2 and CNGA3 express functional channels as homomers. Three additional subunits *CNGA4* (Q8IV77), *CNGB1* (Q14028) and *CNGB3*(Q9NQW8) do not, and are referred to as auxiliary subunits. The subunit composition of the native channels is believed to be as follows. Rod: CNGA1_3_/CNGB1a; Cone: CNGA3_2_/CNGB3_2_; Olfactory neurons: CNGA2_2_/CNGA4/CNGB1b [287, 393, 420, 421, 423].

### Hyperpolarisation‐activated, cyclic nucleotide‐gated (HCN)

The hyperpolarisation‐activated, cyclic nucleotide‐gated (HCN) channels are cation channels that are activated by hyperpolarisation at voltages negative to  ‐50 mV. The cyclic nucleotides cyclic AMP and cyclic GMP directly activate the channels and shift the activation curves of HCN channels to more positive voltages, thereby enhancing channel activity. HCN channels underlie pacemaker currents found in many excitable cells including cardiac cells and neurons [82, 274]. In native cells, these currents have a variety of names, such as *I*_*h*_, *I*_*q*_ and*I*_*f*_. The four known HCN channels have six transmembrane domains and form tetramers. It is believed that the channels can form heteromers with each other, as has been shown for HCN1 and HCN4 [7]. **A standardised nomenclature for HCN channels has been proposed by the NC‐IUPHAR subcommittee on voltage‐gated ion channels** [**138**].

**Table:** 

Nomenclature	HCN1	HCN2	HCN3	HCN4
HGNC, UniProt	*HCN1*, O60741	*HCN2*, Q9UL51	*HCN3*, Q9P1Z3	*HCN4*, Q9Y3Q4
Activators	cyclic AMP>cyclic GMP (both weak)	cyclic AMP>cyclic GMP	–	cyclic AMP>cyclic GMP
Channel blockers	ivabradine (Antagonist) (pIC_50_ 5.7) [‐40mV] [337], ZD7288 (Antagonist) (pIC_50_ 4.7) [‐40mV] [336], Cs^+^ (Antagonist) (pIC_50_ 3.7) [‐40mV] [336]	ivabradine (Antagonist) (pIC_50_ 5.6) [‐40mV] [337] – Mouse, ZD7288 (Antagonist) (pIC_50_ 4.4) [‐40mV] [336], Cs^+^ (Antagonist) (pIC_50_ 3.7) [‐40mV] [336]	ivabradine (Antagonist) (pIC_50_ 5.7) [‐40mV] [337], ZD7288 (Antagonist) (pIC_50_ 4.5) [‐40mV] [336], Cs^+^ (Antagonist) (pIC_50_ 3.8) [‐40mV] [336]	ivabradine (Antagonist) (pIC_50_ 5.7) [‐40mV] [337], ZD7288 (Antagonist) (pIC_50_ 4.7) [‐40mV] [336], Cs^+^ (Antagonist) (pIC_50_ 3.8) [‐40mV] [336]

### Comments

HCN channels are permeable to both Na^+^ and K^+^ ions, with a Na^+^/K^+^ permeability ratio of about 0.2. Functionally, they differ from each other in terms of time constant of activation with HCN1 the fastest, HCN4 the slowest and HCN2 and HCN3 intermediate. The compounds ZD7288 [32] and ivabradine [38] have proven useful in identifying and studying functional HCN channels in native cells. Zatebradine and cilobradine are also useful blocking agents.

### Further Reading

Baruscotti M *et al*. (2010) HCN‐related channelopathies. *Pflugers Arch.* **460**: 405‐15 [PMID:20213494]

Baruscotti M *et al*. (2005) Physiology and pharmacology of the cardiac pacemaker ("funny") current. *Pharmacol. Ther.* **107**: 59‐79 [PMID:15963351]

Biel M *et al*. (2009) Cyclic nucleotide‐gated channels. *Handb Exp Pharmacol* 111‐36 [PMID:19089328]

Biel M *et al*. (2009) Hyperpolarization‐activated cation channels: from genes to function. *Physiol. Rev.* **89**: 847‐85 [PMID:19584315]

Bois P *et al*. (2007) Molecular regulation and pharmacology of pacemaker channels. *Curr. Pharm. Des.* **13**: 2338‐49 [PMID:17692005]

Bradley J *et al*. (2005) Regulation of cyclic nucleotide‐gated channels. *Curr. Opin. Neurobiol.* **15**: 343‐9 [PMID:15922582]

Brown RL *et al*. (2006) The pharmacology of cyclic nucleotide‐gated channels: emerging from the darkness. *Curr. Pharm. Des.* **12**: 3597‐613 [PMID:17073662]

Craven KB *et al*. (2006) CNG and HCN channels: two peas, one pod. *Annu. Rev. Physiol.* **68**: 375‐401 [PMID:16460277]

Cukkemane A *et al*. (2011) Cooperative and uncooperative cyclic‐nucleotide‐gated ion channels. *Trends Biochem. Sci.* **36**: 55‐64 [PMID:20729090]

DiFrancesco D. (2010) The role of the funny current in pacemaker activity. *Circ. Res.* **106**: 434‐46 [PMID:20167941]

Dunlop J *et al*. (2009) Hyperpolarization‐activated cyclic nucleotide‐gated (HCN) channels and pain. *Curr. Pharm. Des.* **15**: 1767‐72 [PMID:19442189]

Hofmann F *et al*. (2005) International Union of Pharmacology. LI. Nomenclature and structure‐function relationships of cyclic nucleotide‐regulated channels. *Pharmacol. Rev.* **57**: 455‐62 [PMID:16382102]

Maher MP *et al*. (2009) HCN channels as targets for drug discovery. *Comb. Chem. High Throughput Screen.* **12**: 64‐72 [PMID:19149492]

Mazzolini M *et al*. (2010) Gating in CNGA1 channels. *Pflugers Arch.* **459**: 547‐55 [PMID:19898862]

Meldrum BS *et al*. (2007) Molecular targets for antiepileptic drug development. *Neurotherapeutics* **4**: 18‐61 [PMID:17199015]

Tardif JC. (2008) Ivabradine: I(f) inhibition in the management of stable angina pectoris and other cardiovascular diseases. *Drugs Today* **44**: 171‐81 [PMID:18536779]

Wahl‐Schott C *et al*. (2009) HCN channels: structure, cellular regulation and physiological function. *Cell. Mol. Life Sci.* **66**: 470‐94 [PMID:18953682]

## Potassium channels

### Overview

Potassium channels are fundamental regulators of excitability. They control the frequency and the shape of action potential waveform, the secretion of hormones and neurotransmitters and cell membrane potential. Their activity may be regulated by voltage, calcium and neurotransmitters (and the signalling pathways they stimulate). They consist of a primary pore‐forming a subunit often associated with auxiliary regulatory subunits. Since there are over 70 different genes encoding K channels *α* subunits in the human genome, it is beyond the scope of this guide to treat each subunit individually. Instead, channels have been grouped into families and subfamilies based on their structural and functional properties. The three main families are the 2TM (two transmembrane domain), 4TM and 6TM families. **A standardised nomenclature for potassium channels has been proposed by the NC‐IUPHAR subcommittees on potassium channels** [**106**, **120**, **191**, **392**].

### Further Reading

Ahern CA *et al*. (2009) Chemical tools for K(+) channel biology. *Biochemistry* **48**: 517‐26 [PMID:19113860]

Bayliss DA *et al*. (2008) Emerging roles for two‐pore‐domain potassium channels and their potential therapeutic impact. *Trends Pharmacol. Sci.* **29**: 566‐75 [PMID:18823665]

Bean BP. (2007) The action potential in mammalian central neurons. *Nat. Rev. Neurosci.* **8**: 451‐65 [PMID:17514198]

Dalby‐Brown W *et al*. (2006) K(v)7 channels: function, pharmacology and channel modulators. *Curr Top Med Chem* **6**: 999‐1023 [PMID:16787276]

Enyedi P *et al*. (2010) Molecular background of leak K+ currents: two‐pore domain potassium channels. *Physiol. Rev.* **90**: 559‐605 [PMID:20393194]

Goldstein SA *et al*. (2005) International Union of Pharmacology. LV. Nomenclature and molecular relationships of two‐P potassium channels. *Pharmacol. Rev.* **57**: 527‐40 [PMID:16382106]

Gutman GA *et al*. (2005) International Union of Pharmacology. LIII. Nomenclature and molecular relationships of voltage‐gated potassium channels. *Pharmacol. Rev.* **57**: 473‐508 [PMID:16382104]

Hancox JC *et al*. (2008) The hERG potassium channel and hERG screening for drug‐induced torsades de pointes. *Pharmacol. Ther.* **119**: 118‐32 [PMID:18616963]

Hansen JB. (2006) Towards selective Kir6.2/SUR1 potassium channel openers, medicinal chemistry and therapeutic perspectives. *Curr. Med. Chem.* **13**: 361‐76 [PMID:16475928]

Honoré E. (2007) The neuronal background K2P channels: focus on TREK1. *Nat. Rev. Neurosci.* **8**: 251‐61 [PMID:17375039]

Jenkinson DH. (2006) Potassium channels–multiplicity and challenges. *Br. J. Pharmacol.* **147 Suppl 1**: S63‐71 [PMID:16402122]

Judge SI *et al*. (2006) Potassium channel blockers in multiple sclerosis: neuronal Kv channels and effects of symptomatic treatment. *Pharmacol. Ther.* **111**: 224‐59 [PMID:16472864]

Kannankeril P *et al*. (2010) Drug‐induced long QT syndrome. *Pharmacol. Rev.* **62**: 760‐81 [PMID:21079043]

Kobayashi T *et al*. (2006) G protein‐activated inwardly rectifying potassium channels as potential therapeutic targets. *Curr. Pharm. Des.* **12**: 4513‐23 [PMID:17168757]

Kubo Y *et al*. (2005) International Union of Pharmacology. LIV. Nomenclature and molecular relationships of inwardly rectifying potassium channels. *Pharmacol. Rev.* **57**: 509‐26 [PMID:16382105]

Lawson K *et al*. (2006) Modulation of potassium channels as a therapeutic approach. *Curr. Pharm. Des.* **12**: 459‐70 [PMID:16472139]

Mannhold R. (2006) Structure‐activity relationships of K(ATP) channel openers. *Curr Top Med Chem* **6**: 1031‐47 [PMID:16787278]

Mathie A *et al*. (2007) Therapeutic potential of neuronal two‐pore domain potassium‐channel modulators. *Curr Opin Investig Drugs* **8**: 555‐62 [PMID:17659475]

Nardi A *et al*. (2008) BK channel modulators: a comprehensive overview. *Curr. Med. Chem.* **15**: 1126‐46 [PMID:18473808]

Pongs O *et al*. (2010) Ancillary subunits associated with voltage‐dependent K+ channels. *Physiol. Rev.* **90**: 755‐96 [PMID:20393197]

Salkoff L *et al*. (2006) High‐conductance potassium channels of the SLO family. *Nat. Rev. Neurosci.* **7**: 921‐31 [PMID:17115074]

Stocker M. (2004) Ca(2+)‐activated K+ channels: molecular determinants and function of the SK family. *Nat. Rev. Neurosci.* **5**: 758‐70 [PMID:15378036]

Takeda M *et al*. (2011) Potassium channels as a potential therapeutic target for trigeminal neuropathic and inflammatory pain. *Mol Pain* **7**: 5 [PMID:21219657]

Trimmer JS *et al*. (2004) Localization of voltage‐gated ion channels in mammalian brain. *Annu. Rev. Physiol.* **66**: 477‐519 [PMID:14977411]

Wang H *et al*. (2007) ATP‐sensitive potassium channel openers and 2,3‐dimethyl‐2‐butylamine derivatives. *Curr. Med. Chem.* **14**: 133‐55 [PMID:17266574]

Weatherall KL *et al*. (2010) Small conductance calcium‐activated potassium channels: from structure to function. *Prog. Neurobiol.* **91**: 242‐55 [PMID:20359520]

Wei AD *et al*. (2005) International Union of Pharmacology. LII. Nomenclature and molecular relationships of calcium‐activated potassium channels. *Pharmacol. Rev.* **57**: 463‐72 [PMID:16382103]

Wickenden AD *et al*. (2009) Kv7 channels as targets for the treatment of pain. *Curr. Pharm. Des.* **15**: 1773‐98 [PMID:19442190]

Witchel HJ. (2007) The hERG potassium channel as a therapeutic target. *Expert Opin. Ther. Targets* **11**: 321‐36 [PMID:17298291]

Wulff H *et al*. (2009) Voltage‐gated potassium channels as therapeutic targets. *Nat Rev Drug Discov* **8**: 982‐1001 [PMID:19949402]

## Calcium‐activated potassium channels

### Overview

The 6TM family of K channels comprises the voltage‐gated K_*V*_subfamilies, the KCNQ subfamily, the EAG subfamily (which includes herg channels), the Ca^2+^‐activated Slo subfamily (actually with 6 or 7TM) and the Ca^2+^‐activated SK subfamily. As for the 2TM family, the pore‐forming a subunits form tetramers and heteromeric channels may be formed within subfamilies (*e.g.* K_*V*_1.1 with K_*V*_1.2; KCNQ2 with KCNQ3).

**Table:** 

Nomenclature	K_*Ca*_1.1	K_*Ca*_2.1	K_*Ca*_2.2	K_*Ca*_2.3
HGNC, UniProt	*KCNMA1*, Q12791	*KCNN1*, Q92952	*KCNN2*, Q9H2S1	*KCNN3*, Q9UGI6
Functional Characteristics	Maxi K_*Ca*_	SK_*Ca*_	SK_*Ca*_	SK_*Ca*_
Activators	NS004, NS1619	EBIO (Agonist) Concentration range: 2 × 10^−3^M [‐80mV] [284, 390], NS309 (Agonist) Concentration range: 3 × 10^−8^M‐1 × 10^−7^M [‐90mV] [341, 390]	NS309 (Agonist) (pEC_50_ 6.2) Concentration range: 3 × 10^−8^M‐1 × 10^−7^M [‐90mV – ‐50mV] [283, 341, 390], EBIO (Agonist) (pEC_50_ 3.3) [‐50mV] [283, 390], EBIO (Agonist) (pEC_50_ 3) Concentration range: 2 × 10^−3^M [‐100mV] [44, 284] – Rat	EBIO (Agonist) (pEC_50_ 3.8) [‐160mV – ‐120mV] [390, 398], NS309 (Agonist) Concentration range: 3 × 10^−8^M [‐90mV] [341, 390]
Inhibitors	charybdotoxin, iberiotoxin, tetraethylammonium	–	–	–
Channel blockers	paxilline (Antagonist) (p*K*_*i*_ 8.7) [0mV] [316] – Mouse	UCL1684 (Antagonist) (pIC_50_ 9.1) [‐80mV] [340, 390], apamin (Antagonist) (pIC_50_ 7.9–8.5, median 8.1) [‐80mV] [323, 338, 340], tetraethylammonium (Antagonist) (pIC_50_ 2.7) [390]	UCL1684 (Antagonist) (pIC_50_ 9.6) [‐40mV] [94, 390], apamin (Antagonist) (p*K*_*d*_ 9.4) [‐80mV] [161], tetraethylammonium (Antagonist) (pIC_50_ 2.7) [390]	apamin (Antagonist) (pIC_50_ 7.9–9.1) [‐160mV – ‐100mV] [358, 398], UCL1684 (Antagonist) (pIC_50_ 8–9) [‐80mV] [94, 390], tetraethylammonium (Antagonist) (pIC_50_ 2.7) [390]
Comments	–	The rat isoform does not form functional channels when expressed alone in cell lines. N‐ or C‐terminal chimeric constructs permit functional channels that are insensitive to apamin [390]. Heteromeric channels are formed between K_*Ca*_2.1 and 2.2 subunits that show intermediate sensitivity to apamin [63].	–	–

**Table:** 

Nomenclature	K_*Ca*_3.1	K_*Na*_1.1	K_*Na*_1.2	K_*Ca*_5.1
HGNC, UniProt	*KCNN4*, O15554	*KCNT1*, Q5JUK3	*KCNT2*, Q6UVM3	*KCNU1*, A8MYU2
Functional Characteristics	IK_*Ca*_	K_*Na*_	K_*Na*_	Sperm pH‐regulated K^+^ current, KSPER
Activators	NS309 (Agonist) (pEC_50_ 8) [‐90mV] [341, 390], SKA‐121 (Agonist) (pEC_50_ 7) [67], EBIO (Agonist) (pEC_50_ 4.1–4.5) [‐100mV – ‐50mV] [284, 346, 390]	bithionol (Agonist) (pEC_50_ 5–6) [414] – Rat, niclosamide (Agonist) (pEC_50_ 5.5) [30], loxapine (Agonist) (pEC_50_ 5.4) [30]	niflumic acid (Agonist) [71]	–
Gating inhibitors	–	bepridil (Antagonist) (pIC_50_ 5–6) [9, 27, 414] – Rat	–	–
Channel blockers	charybdotoxin (Inhibition) (pIC_50_ 7.6–8.7) [153, 157], TRAM‐34 (Inhibition) (p*K*_*d*_ 7.6–8) [193, 403]	quinidine (Antagonist) (pIC_50_ 4) [414] – Rat	Ba^2+^ (Inhibition) (pIC_50_ 3) [27], quinidine (Inhibition) Concentration range: 1 × 10^−3^M [27] – Rat	tetraethylammonium (pEC_50_ 2.3) [319, 355] – Mouse, quinidine [355] – Mouse

## Inwardly rectifying potassium channels

### Overview

The 2TM domain family of K channels are also known as the inward‐rectifier K channel family. This family includes the strong inward‐rectifier K channels (K_*ir*_2.x), the G‐protein‐activated inward‐rectifier K channels (K_*ir*_3.x) and the ATP‐sensitive K channels (K_*ir*_6.x, which combine with sulphonylurea receptors (SUR)). The pore‐forming a subunits form tetramers, and heteromeric channels may be formed within subfamilies (*e.g.* K_*ir*_3.2 with K_*ir*_3.3).

**Table:** 

Nomenclature	K_*ir*_1.1	K_*ir*_2.1	K_*ir*_2.2
HGNC, UniProt	*KCNJ1*, P48048	*KCNJ2*, P63252	*KCNJ12*, Q14500
Ion Selectivity and Conductance	NH_4_^+^ [62pS] > K^+^ [38. pS] > Tl^+^ [21pS] > Rb^+^ [15pS] (Rat) [57, 134]	–	–
Functional Characteristics	K_*ir*_1.1 is weakly inwardly rectifying, as compared to classical (strong) inward rectifiers.	IK_1_ in heart, ‘strong’ inward‐rectifier current	IK_1_in heart, ‘strong’ inward‐rectifier current
Endogenous activators	–	PIP_2_ (Agonist) Concentration range: 1 × 10^−5^M‐5 × 10^−5^M [‐30mV] [142, 307, 334] – Mouse	–
Endogenous inhibitors	–	–	Intracellular Mg^2+^ (pIC_50_ 5) [40mV] [413]
Gating inhibitors	–	–	Ba^2+^ (Antagonist) Concentration range: 5 × 10^−5^M [‐150mV – ‐50mV] [349] – Mouse, Cs^+^ (Antagonist) Concentration range: 5 × 10^−6^M‐5 × 10^−5^M [‐150mV – ‐50mV] [349] – Mouse
Endogenous channel blockers	–	spermine (Antagonist) (p*K*_*d*_ 9.1) [*voltage dependent* 40mV] [150, 415] – Mouse, spermidine (Antagonist) (p*K*_*d*_ 8.1) [*voltage dependent* 40mV] [415] – Mouse, putrescine (Antagonist) (p*K*_*d*_ 5.1) [*voltage dependent* 40mV] [150, 415] – Mouse, Intracellular Mg^2+^ (Antagonist) (p*K*_*d*_ 4.8) [*voltage dependent* 40mV] [415] – Mouse	–
Channel blockers	tertiapin‐Q (Inhibition) (pIC_50_ 8.9) [156], Ba^2+^ (Antagonist) (pIC_50_ 2.3–4.2) Concentration range: 1 × 10^−4^M [*voltage dependent* 0mV – ‐100mV] [134, 424] – Rat, Cs^+^ (Antagonist) (pIC_50_ 2.9) [*voltage dependent* ‐120mV] [424] – Rat	Ba^2+^ (Antagonist) (p*K*_*d*_ 3.9–5.6) Concentration range: 1 × 10^−6^M‐1 × 10^−4^M [*voltage dependent* 0mV – ‐80mV] [6] – Mouse, Cs^+^ (Antagonist) (p*K*_*d*_ 1.3–4) Concentration range: 3 × 10^−5^M‐3 × 10^−4^M [*voltage dependent* 0mV – ‐102mV] [3] – Mouse	–
Comments	–	K_*ir*_2.1 is also inhibited by intracellular polyamines	K_*ir*_2.2 is also inhibited by intracellular polyamines

**Table:** 

Nomenclature	K_*ir*_2.3	K_*ir*_2.4	K_*ir*_3.1	K_*ir*_3.2
HGNC, UniProt	*KCNJ4*, P48050	*KCNJ14*, Q9UNX9	*KCNJ3*, P48549	*KCNJ6*, P48051
Functional Characteristics	IK_1_ in heart, ‘strong’ inward‐rectifier current	IK_1_ in heart, ‘strong’ inward‐rectifier current	G‐protein‐activated inward‐rectifier current	G‐protein‐activated inward‐rectifier current
Endogenous activators	–	–	PIP_2_ (Agonist) (p*K*_*d*_ 6.3) Concentration range: 5 × 10^−5^M [physiological voltage] [142] – Unknown	PIP_2_ (Agonist) (p*K*_*d*_ 6.3) Concentration range: 5 × 10^−5^M [physiological voltage] [142] – Unknown
Endogenous inhibitors	–	Intracellular Mg^2+^	–	–
Gating inhibitors	–	–	–	pimozide (Antagonist) (pEC_50_ 5.5) [‐70mV] [180] – Mouse
Endogenous channel blockers	Intracellular Mg^2+^ (Antagonist) (p*K*_*d*_ 5) [*voltage dependent* 50mV] [222], putrescine (Antagonist) Concentration range: 5 × 10^−5^M‐1 × 10^−3^M [‐80mV – 80mV] [222], spermidine (Antagonist) Concentration range: 2.5 × 10^−5^M‐1 × 10^−3^M [‐80mV – 80mV] [222], spermine (Antagonist) Concentration range: 5 × 10^−5^M‐1 × 10^−3^M [‐80mV – 80mV] [222]	–	–	–
Channel blockers	Ba^2+^ (Antagonist) (pIC_50_ 5) Concentration range: 3 × 10^−6^M‐5 × 10^−4^M [‐60mV] [233, 296, 356], Cs^+^ (Antagonist) (p*K*_*i*_ 1.3–4.5) Concentration range: 3 × 10^−6^M‐3 × 10^−4^M [0mV – ‐130mV] [233]	Cs^+^ (Antagonist) (p*K*_*d*_ 3–4.1) [*voltage dependent* ‐60mV – ‐100mV] [143], Ba^2+^ (Antagonist) (p*K*_*d*_ 3.3) [*voltage dependent* 0mV] [143]	tertiapin‐Q (Antagonist) (pIC_50_ 7.9) [156], Ba^2+^ (Antagonist) (pIC_50_ 4.7) [73] – Rat	desipramine (Antagonist) (pIC_50_ 4.4) [‐70mV] [181] – Mouse
Comments	K_*ir*_2.3 is also inhibited by intracellular polyamines	K_*ir*_2.4 is also inhibited by intracellular polyamines	K_*ir*_3.1 is also activated by G_*βγ*_. K_*ir*_3.1 is not functional alone. The functional expression of K_*ir*_3.1 in *Xenopus oocytes* requires coassembly with the endogenous *Xenopus* K_*ir*_3.5 subunit. The major functional assembly in the heart is the K_*ir*_3.1/3.4 heteromultimer, while in the brain it is K_*ir*_3.1/3.2, K_*ir*_3.1/3.3 and K_*ir*_3.2/3.3.	K_*ir*_3.2 is also activated by G_*βγ*_. K_*ir*_3.2 forms functional heteromers with K_*ir*_3.1/3.3.

**Table:** 

Nomenclature	K_*ir*_3.3	K_*ir*_3.4	K_*ir*_4.1	K_*ir*_4.2
HGNC, UniProt	*KCNJ9*, Q92806	*KCNJ5*, P48544	*KCNJ10*, P78508	*KCNJ15*, Q99712
Functional Characteristics	G‐protein‐activated inward‐rectifier current	G‐protein‐activated inward‐rectifier current	Inward‐rectifier current	Inward‐rectifier current
Endogenous activators	PIP_2_ [129]	PIP_2_ [20, 129]	–	–
Channel blockers	–	tertiapin‐Q (Antagonist) (pIC_50_ 7.9) [156]	Ba^2+^ (Antagonist) Concentration range: 3 × 10^−6^M‐1 × 10^−3^M [‐160mV – 60mV] [185, 351, 354] – Rat, Cs^+^ (Antagonist) Concentration range: 3 × 10^−5^M‐3 × 10^−4^M [‐160mV – 50mV] [351] – Rat	Ba^2+^ (Antagonist) Concentration range: 1 × 10^−5^M‐1 × 10^−4^M [‐120mV – 100mV] [282] – Mouse, Cs^+^ (Antagonist) Concentration range: 1 × 10^−5^M‐1 × 10^−4^M [‐120mV – 100mV] [282] – Mouse
Comments	K_*ir*_3.3 is also activated by G_*βγ*_	K_*ir*_3.4 is also activated by G_*βγ*_	–	–

**Table:** 

Nomenclature	K_*ir*_5.1	K_*ir*_6.1	K_*ir*_6.2	K_*ir*_7.1
HGNC, UniProt	*KCNJ16*, Q9NPI9	*KCNJ8*, Q15842	*KCNJ11*, Q14654	*KCNJ13*, O60928
Associated subunits	–	SUR1, SUR2A, SUR2B	SUR1, SUR2A, SUR2B	–
Functional Characteristics	Weakly inwardly rectifying	ATP‐sensitive, inward‐rectifier current	ATP‐sensitive, inward‐rectifier current	Inward‐rectifier current
Activators	–	cromakalim, diazoxide (Agonist) Concentration range: 2 × 10^−4^M [‐60mV] [411] – Mouse, minoxidil, nicorandil (Agonist) Concentration range: 3 × 10^−4^M [‐60mV – 60mV] [411] – Mouse	diazoxide (Agonist) (pEC_50_ 4.2) [physiological voltage] [146] – Mouse, cromakalim (Agonist) Concentration range: 3 × 10^−5^M [‐60mV] [147] – Mouse, minoxidil, nicorandil	–
Inhibitors	–	glibenclamide, tolbutamide	glibenclamide, tolbutamide	–
Channel blockers	Ba^2+^ (Antagonist) Concentration range: 3 × 10^−3^M [‐120mV – 20mV] [353] – Rat	–	–	Ba^2+^ (Antagonist) (p*K*_*i*_ 3.2) [*voltage dependent* ‐100mV] [90, 190, 192, 277], Cs^+^ (Antagonist) (p*K*_*i*_ 1.6) [*voltage dependent* ‐100mV] [90, 190, 277]

## Two‐P potassium channels

### Overview

The 4TM family of K channels are thought to underlie many background K currents in native cells. They are open at all voltages and regulated by a wide array of neurotransmitters and biochemical mediators. The primary pore‐forming *α*‐subunit contains two pore domains (indeed, they are often referred to as two‐pore domain K channels or K2P) and so it is envisaged that they form functional dimers rather than the usual K channel tetramers. There is some evidence that they can form heterodimers within subfamilies (*e.g.* K_2*P*_3.1 with K_2*P*_9.1). There is no current, clear, consensus on nomenclature of 4TM K channels, nor on the division into subfamilies [106]. The suggested division into subfamilies, below, is based on similarities in both structural and functional properties within subfamilies.

**Table:** 

Nomenclature	K_2*P*_1.1	K_2*P*_2.1	K_2*P*_3.1	K_2*P*_4.1	K_2*P*_5.1
HGNC, UniProt	*KCNK1*, O00180	*KCNK2*, O95069	*KCNK3*, O14649	*KCNK4*, Q9NYG8	*KCNK5*, O95279
Functional Characteristics	Background current	Background current	Background current. Knock‐out of the *kcnk3* gene leads to a prolonged QT interval in mice [77].	Background current	Background current
Endogenous activators	–	arachidonic acid (pEC_50_ 5)	–	arachidonic acid (Positive) Concentration range: 5 × 10^−6^M‐5 × 10^−5^M [168] – Rat	–
Activators	–	halothane, riluzole	halothane (Positive) (pEC_50_ 3) Concentration range: 1 × 10^−3^M [389] – Rat	riluzole (Positive) Concentration range: 3 × 10^−6^M‐1 × 10^−4^M [88]	–
Channel blockers	–	–	anandamide (Inhibition) (pIC_50_ 5.6) [230]	–	–
Comments	K_2*P*_1.1 is inhibited by acid pH_*o*_	K_2*P*_2.1 is also activated by stretch, heat and acid pH_*i*_	K_2*P*_3.1 is also activated by alkaline pH_*o*_ and inhibited by acid pH_*o*_	K_2*P*_4.1 is also activated by heat, acid pH_*i*_, and membrane stretch	K_2*P*_5.1 is activated by alkaline pH_*o*_

**Table:** 

Nomenclature	K_2*P*_6.1	K_2*P*_7.1	K_2*P*_9.1	K_2*P*_10.1	K_2*P*_12.1
HGNC, UniProt	*KCNK6*, Q9Y257	*KCNK7*, Q9Y2U2	*KCNK9*, Q9NPC2	*KCNK10*, P57789	*KCNK12*, Q9HB15
Functional Characteristics	Unknown	Unknown	Background current	Background current	Unknown
Endogenous activators	–	–	–	arachidonic acid [203]	–
Activators	–	–	halothane	halothane, riluzole	–
Inhibitors	–	–	anandamide, ruthenium red	–	halothane
Comments	–	–	K_2*P*_9.1 is also inhibited by acid pH_*o*_	K_2*P*_10.1 is also activated by heat, acid pH_*i*_, and membrane stretch	–

**Table:** 

Nomenclature	K_2*P*_13.1	K_2*P*_15.1	K_2*P*_16.1	K_2*P*_17.1	K_2*P*_18.1
HGNC, UniProt	*KCNK13*, Q9HB14	*KCNK15*, Q9H427	*KCNK16*, Q96T55	*KCNK17*, Q96T54	*KCNK18*, Q7Z418
Functional Characteristics	Background current	Unknown	Background current	Background current	Background current
Endogenous inhibitors	–	–	–	–	arachidonic acid
Inhibitors	halothane	–	–	–	–
Comments	–	–	K_2*P*_16.1 is activated by alkaline pH_*o*_	K_2*P*_17.1 is activated by alkaline pH_*o*_	–

### Comments

The K_2*P*_7.1, K_2*P*_15.1 and K_2*P*_12.1 subtypes, when expressed in isolation, are nonfunctional. All 4TM channels are insensitive to the classical potassium channel blockers tetraethylammonium and fampridine, but are blocked to varying degrees by Ba^2+^ ions.

## Voltage‐gated potassium channels

### Overview

The 6TM family of K channels comprises the voltage‐gated K_*V*_subfamilies, the KCNQ subfamily, the EAG subfamily (which includes herg channels), the Ca^2+^‐activated Slo subfamily (actually with 6 or 7TM) and the Ca^2+^‐activated SK subfamily. As for the 2TM family, the pore‐forming a subunits form tetramers and heteromeric channels may be formed within subfamilies (*e.g.* K_*V*_1.1 with K_*V*_1.2; KCNQ2 with KCNQ3).

**Table:** 

Nomenclature	K_*v*_1.1	K_*v*_1.2	K_*v*_1.3	K_*v*_1.4	K_*v*_1.5
HGNC, UniProt	*KCNA1*, Q09470	*KCNA2*, P16389	*KCNA3*, P22001	*KCNA4*, P22459	*KCNA5*, P22460
Associated subunits	K_*v*_1.2, K_*v*_1.4, K_*v*_ *β*1 and K_*v*_ *β*2 [68]	K_*v*_1.1, K_*v*_1.4, K_*v*_ *β*1 and K_*v*_ *β*2 [68]	K_*v*_1.1, K_*v*_1.2, K_*v*_1.4, K_*v*_1.6 , K_*v*_ *β*1 and K_*v*_ *β*2 [68]	K_*v*_1.1, K_*v*_1.2, K_*v*_ *β*1 and K_*v*_ *β*2 [68]	K_*v*_ *β*1 and K_*v*_ *β*2
Functional Characteristics	K_*V*_	K_*V*_	K_*V*_	K_*A*_	K_*V*_
Channel blockers	*α*‐dendrotoxin (pEC_50_ 7.7–9) [113, 144] – Rat, margatoxin (Inhibition) (pIC_50_ 8.4) [19], tetraethylammonium (Inhibition) (p*K*_*d*_ 3.5) [113] – Mouse	margatoxin (Inhibition) (pIC_50_ 11.2) [19], *α*‐dendrotoxin (pIC_50_ 7.8–9.4) [113, 144] – Rat, noxiustoxin (p*K*_*d*_ 8.7) [113] – Rat	margatoxin (pIC_50_ 10–10.3) [100, 103], noxiustoxin (p*K*_*d*_ 9) [113] – Mouse, tetraethylammonium (moderate) (p*K*_*d*_ 2) [113] – Mouse	fampridine (pIC_50_ 1.9) [344] – Rat	–
Selective channel blockers	–	–	correolide (pIC_50_ 7.1) [95]	–	–

**Table:** 

Nomenclature	K_*v*_1.6	K_*v*_1.7	K_*v*_1.8	K_*v*_2.1	K_*v*_2.2
HGNC, UniProt	*KCNA6*, P17658	*KCNA7*, Q96RP8	*KCNA10*, Q16322	*KCNB1*, Q14721	*KCNB2*, Q92953
Associated subunits	K_*v*_ *β*1 and K_*v*_ *β*2	K_*v*_ *β*1 and K_*v*_ *β*2	K_*v*_ *β*1 and K_*v*_ *β*2	K_*v*_5.1, K_*v*_6.1‐6.4, K_*v*_8.1‐8.2 and K_*v*_9.1‐9.3	K_*v*_5.1, K_*v*_6.1‐6.4, K_*v*_8.1‐8.2 and K_*v*_9.1‐9.3
Functional Characteristics	K_*V*_	K_*V*_	K_*V*_	K_*V*_	–
Channel blockers	*α*‐dendrotoxin (pIC_50_ 7.7) [114], tetraethylammonium (pIC_50_ 2.2) [114]	fampridine (pIC_50_ 3.6) [162] – Mouse	fampridine (pIC_50_ 2.8) [195]	tetraethylammonium (Pore blocker) (pIC_50_ 2) [127] – Rat	fampridine (pIC_50_ 2.8) [318], tetraethylammonium (pIC_50_ 2.6) [318]

**Table:** 

Nomenclature	K_*v*_3.1	K_*v*_3.2	K_*v*_3.3	K_*v*_3.4	K_*v*_4.1
HGNC, UniProt	*KCNC1*, P48547	*KCNC2*, Q96PR1	*KCNC3*, Q14003	*KCNC4*, Q03721	*KCND1*, Q9NSA2
Associated subunits	–	–	–	MiRP2 is an associated subunit for K_*v*_3.4	KChIP and KChAP
Functional Characteristics	K_*V*_	K_*V*_	K_*A*_	K_*A*_	K_*A*_
Channel blockers	fampridine (pIC_50_ 4.5) [113] – Mouse, tetraethylammonium (pIC_50_ 3.7) [113] – Mouse	fampridine (pIC_50_ 4.6) [210] – Rat, tetraethylammonium (pIC_50_ 4.2) [210] – Rat	tetraethylammonium (pIC_50_ 3.9) [367] – Rat	tetraethylammonium (pIC_50_ 3.5) [309, 321] – Rat	fampridine (pIC_50_ 2) [149]
Selective channel blockers	–	–	–	sea anemone toxin BDS‐I (pIC_50_ 7.3) [84] – Rat	–

**Table:** 

Nomenclature	K_*v*_4.2	K_*v*_4.3	K_*v*_5.1	K_*v*_6.1	K_*v*_6.2	K_*v*_6.3	K_*v*_6.4
HGNC, UniProt	*KCND2*, Q9NZV8	*KCND3*, Q9UK17	*KCNF1*, Q9H3M0	*KCNG1*, Q9UIX4	*KCNG2*, Q9UJ96	*KCNG3*, Q8TAE7	*KCNG4*, Q8TDN1
Associated subunits	KChIP and KChAP	KChIP and KChAP	–	–	–	–	–
Functional Characteristics	K_*A*_	K_*A*_	–	–	–	–	–

**Table:** 

Nomenclature	K_*v*_7.1	K_*v*_7.2	K_*v*_7.3	K_*v*_7.4	K_*v*_7.5
HGNC, UniProt	*KCNQ1*, P51787	*KCNQ2*, O43526	*KCNQ3*, O43525	*KCNQ4*, P56696	*KCNQ5*, Q9NR82
Functional Characteristics	cardiac IK_5_	M current	M current	–	–
Activators	–	retigabine (pEC_50_ 5.6) [357]	retigabine (pEC_50_ 6.2) [357]	retigabine (pEC_50_ 5.2) [357]	retigabine (pEC_50_ 5) [89]
Inhibitors	linopirdine (pIC_50_ 4.4) [271] – Mouse	–	linopirdine (pIC_50_ 5.4) [385] – Rat	–	–
Channel blockers	XE991 (Antagonist) (p*K*_*d*_ 6.1) [384]	XE991 (pIC_50_ 6.2) [385], linopirdine (pIC_50_ 5.3) [385], tetraethylammonium (pIC_50_ 3.5–3.9) [121, 394]	–	XE991 (pIC_50_ 5.3) [348], linopirdine (pIC_50_ 4.9) [348], tetraethylammonium (pIC_50_ 1.3) [14]	linopirdine (p*K*_*d*_ 4.8) [202]
(Sub)family‐selective channel blockers	–	–	–	–	XE991 (pIC_50_ 4.2) [320]

**Table:** 

Nomenclature	K_*v*_8.1	K_*v*_8.2	K_*v*_9.1	K_*v*_9.2	K_*v*_9.3	K_*v*_10.1	K_*v*_10.2
HGNC, UniProt	*KCNV1*, Q6PIU1	*KCNV2*, Q8TDN2	*KCNS1*, Q96KK3	*KCNS2*, Q9ULS6	*KCNS3*, Q9BQ31	*KCNH1*, O95259	*KCNH5*, Q8NCM2

**Table:** 

Nomenclature	K_*v*_11.1	K_*v*_11.2	K_*v*_11.3	K_*v*_12.1	K_*v*_12.2	K_*v*_12.3
HGNC, UniProt	*KCNH2*, Q12809	*KCNH6*, Q9H252	*KCNH7*, Q9NS40	*KCNH8*, Q96L42	*KCNH3*, Q9ULD8	*KCNH4*, Q9UQ05
Associated subunits	minK (KCNE1) and MiRP1 (KCNE2)	minK (KCNE1)	minK (KCNE1)	minK (KCNE1)	minK (KCNE1) and MiRP2 (KCNE3)	–
Functional Characteristics	cardiac IK_*R*_	–	–	–	–	–
Channel blockers	astemizole (pIC_50_ 9) [426], terfenadine (pIC_50_ 7.3) [303], disopyramide (Inhibition) (pIC_50_ 4) [167]	–	–	–	–	–
(Sub)family‐selective channel blockers	E4031 (pIC_50_ 8.1) [425]	–	–	–	–	–
Selective channel blockers	dofetilide (Inhibition) (p*K*_*i*_ 8.2) [328], ibutilide (pIC_50_ 7.6–8) [167, 290]	–	–	–	–	–
Comments	RPR260243 is an activator of K_*v*_11.1 [163].	–	–	–	–	–

## Transient Receptor Potential channels

### Overview

The TRP superfamily of channels (**nomenclature as agreed by NC‐IUPHAR**[**65**, **402**]), whose founder member is the *Drosophila* Trp channel, exists in mammals as six families; TRPC, TRPM, TRPV, TRPA, TRPP and TRPML based on amino acid homologies. TRP subunits contain six putative transmembrane domains and assemble as homo‐ or hetero‐tetramers to form cation selective channels with diverse modes of activation and varied permeation properties (reviewed by [273]). Established, or potential, physiological functions of the individual members of the TRP families are discussed in detail in the recommended reviews and a compilation edited by Islam [151]. The established, or potential, involvement of TRP channels in disease is reviewed in [174, 258] and [260], together with a special edition of *Biochemica et Biophysica Acta* on the subject [258]. The pharmacology of most TRP channels is poorly developed [402]. Broad spectrum agents are listed in the tables along with more selective, or recently recognised, ligands that are flagged by the inclusion of a primary reference. Most TRP channels are regulated by phosphoinostides such as PtIns(4,5)P_2_and IP_3_ although the effects reported are often complex, occasionally contradictory, and likely to be dependent upon experimental conditions, such as intracellular ATP levels (reviewed by [261, 310, 372]). Such regulation is generally not included in the tables. When thermosensitivity is mentioned, it refers specifically to a high Q10 of gating, often in the range of 10‐30, but does not necessarily imply that the channel's function is to act as a 'hot' or 'cold' sensor. In general, the search for TRP activators has led to many claims for temperature sensing, mechanosensation, and lipid sensing. All proteins are of course sensitive to energies of binding, mechanical force, and temperature, but the issue is whether the proposed input is within a physiologically relevant range resulting in a response.

### TRPA (ankyrin) family

TRPA1 is the sole mammalian member of this group (reviewed by [101]). TRPA1 activation of sensory neurons contribute to nociception [158, 238, 339]. Pungent chemicals such as mustard oil (AITC), allicin, and cinnamaldehyde activate TRPA1 by modification of free thiol groups of cysteine side chains, especially those located in its amino terminus [21, 133, 226, 228]. Alkenals with *α*, *β*‐unsaturated bonds, such as propenal (acrolein), butenal (crotylaldehyde), and 2‐pentenal can react with free thiols *via* Michael addition and can activate TRPA1. However, potency appears to weaken as carbon chain length increases [12, 21]. Covalent modification leads to sustained activation of TRPA1. Chemicals including carvacrol, menthol, and local anesthetics reversibly activate TRPA1 by non‐covalent binding [164, 201, 407, 408]. TRPA1 is not mechanosensitive under physiological conditions, but can be activated by cold temperatures [165, 429]. The electron cryo‐EM structure of TRPA1 [279] indicates that it is a 6‐TM homotetramer. Each subunit of the channel contains two short ‘pore helices’ pointing into the ion selectivity filter, which is big enough to allow permeation of partially hydrated Ca^2+^ ions. A coiled‐coil domain in the carboxy‐terminal region forms the cytoplasmic stalk of the channel, and is surrounded by 16 ankyrin repeat domains, which are speculated to interdigitate with an overlying helix‐turn‐helix and putative *β*‐sheet domain containing cysteine residues targeted by electrophilic TRPA1 agonists. The TRP domain, a helix at the base of S6, runs perpendicular to the pore helices suspended above the ankyrin repeats below, where it may contribute to regulation of the lower pore. The coiled‐coil stalk mediates bundling of the four subunits through interactions between predicted *α*‐helices at the base of the channel.

### TRPC (canonical) family

Members of the TRPC subfamily (reviewed by [2, 8, 25, 29, 99, 172, 278, 298]) fall into the subgroups outlined below. TRPC2 (not tabulated) is a pseudogene in man. It is generally accepted that all TRPC channels are activated downstream of G_*q*/11_‐coupled receptors, or receptor tyrosine kinases (reviewed by [294, 364, 402]). A comprehensive listing of G‐protein coupled receptors that activate TRPC channels is given in [2]. Hetero‐oligomeric complexes of TRPC channels and their association with proteins to form signalling complexes are detailed in [8] and [173]. TRPC channels have frequently been proposed to act as store‐operated channels (SOCs) (or compenents of mulimeric complexes that form SOCs), activated by depletion of intracellular calcium stores (reviewed by [8, 56, 285, 295, 315, 416]), However, the weight of the evidence is that they are not directly gated by conventional store‐operated mechanisms, as established for Stim‐gated Orai channels. TRPC channels are not mechanically gated in physiologically relevant ranges of force. All members of the TRPC family are blocked by 2‐APB and SKF96365[124, 125]. Activation of TRPC channels by lipids is discussed by [25].

### TRPC1/C4/C5 subgroup

TRPC4/C5 may be distinguished from other TRP channels by their potentiation by micromolar concentrations of La^3+^.

### TRPC3/C6/C7 subgroup

All members are activated by diacylglycerol independent of protein kinase C stimulation [125].

### TRPM (melastatin) family

Members of the TRPM subfamily (reviewed by [97, 124, 285, 422]) fall into the five subgroups outlined below.

### TRPM1/M3 subgroup

TRPM1 exists as five splice variants and is involved in normal melanocyte pigmentation [268] and is also a visual transduction channel in retinal ON bipolar cells [183]. TRPM3 (reviewed by [270]) exists as multiple splice variants four of which (mTRPM3*α*1, mTRPM3*α*2, hTRPM3a and hTRPM3_1325_) have been characterised and found to differ significantly in their biophysical properties. TRPM3 may contribute to the detection of noxious heat [376].

### TRPM2

TRPM2 is activated under conditions of oxidative stress (reviewed by [412]). Numerous splice variants of TRPM2 exist which differ in their activation mechanisms [87]. The C‐terminal domain contains a TRP motif, a coiled‐coil region, and an enzymatic NUDT9 homologous domain. TRPM2 appears not to be activated by NAD, NAAD, or NAADP, but is directly activated by ADPRP (adenosine‐5'‐O‐disphosphoribose phosphate) [365].

### TRPM4/5 subgroup

TRPM4 and TRPM5 have the distinction within all TRP channels of being impermeable to Ca^2+^[402]. A splice variant of TRPM4 (*i.e.*TRPM4b) and TRPM5 are molecular candidates for endogenous calcium‐activated cation (CAN) channels [115]. TRPM4 has been shown to be an important regulator of Ca^2+^ entry in to mast cells [368] and dendritic cell migration [18]. TRPM5 in taste receptor cells of the tongue appears essential for the transduction of sweet, amino acid and bitter stimuli [212].

### TRPM6/7 subgroup

TRPM6 and 7 combine channel and enzymatic activities (‘chanzymes’). These channels have the unusual property of permeation by divalent (Ca^2+^, Mg^2+^, Zn^2+^) and monovalent cations, high single channel conductances, but overall extremely small inward conductance when expressed to the plasma membrane. They are inhibited by internal Mg^2+^ at 0.6 mM, around the free level of Mg^2+^ in cells. Whether they contribute to Mg^2+^ homeostasis is a contentious issue. When either gene is deleted in mice, the result is embryonic lethality. The C‐terminal kinase region is cleaved under unknown stimuli, and the kinase phosphorylates nuclear histones.

### TRPM8

Is a channel activated by cooling and pharmacological agents evoking a ‘cool’ sensation and participates in the thermosensation of cold temperatures [23, 66, 81] reviewed by [179, 220, 248, 373].

### TRPML (mucolipin) family

The TRPML family [297, 300, 417] consists of three mammalian members (TRPML1‐3). TRPML channels are probably restricted to intracellular vesicles and mutations in the gene (*MCOLN1*) encoding TRPML1 (mucolipin‐1) are one cause of the neurodegenerative disorder mucolipidosis type IV (MLIV) in man. TRPML1 is a cation selective ion channel that is important for sorting/transport of endosomes in the late endocytotic pathway and specifically fusion between late endosome‐lysosome hybrid vesicles. TRPML3 is important for hair cell maturation, stereocilia maturation and intracellular vesicle transport. A naturally occurring gain of function mutation in TRPML3 (*i.e.* A419P) results in the varitint waddler (V*a*) mouse phenotype (reviewed by [262, 300]).

### TRPP (polycystin) family

The TRPP family (reviewed by [78, 80, 104, 137, 399]) or PKD2 family is comprised of PKD2, PKD2L1 and PKD2L2, which have been renamed TRPP1, TRPP2 and TRPP3, respectively [402]. They are clearly distinct from the PKD1 family, whose function is unknown. Although still being sorted out, TRPP family members appear to be 6TM spanning nonselective cation channels.

### TRPV (vanilloid) family

Members of the TRPV family (reviewed by [369]) can broadly be divided into the non‐selective cation channels, TRPV1‐4 and the more calcium selective channels TRPV5 and TRPV6.

### TRPV1‐V4 subfamily

TRPV1 is involved in the development of thermal hyperalgesia following inflammation and may contribute to the detection of noxius heat (reviewed by [293, 335, 347]). Numerous splice variants of TRPV1 have been described, some of which modulate the activity of TRPV1, or act in a dominant negative manner when co‐expressed with TRPV1 [322]. The pharmacology of TRPV1 channels is discussed in detail in [117] and [375]. TRPV2 is probably not a thermosensor in man [275], but has recently been implicated in innate immunity [214]. TRPV3 and TRPV4 are both thermosensitive. There are claims that TRPV4 is also mechanosensitive, but this has not been established to be within a physiological range in a native environment [43, 209].

### TRPV5/V6 subfamily

Under physiological conditions, TRPV5 and TRPV6 are calcium selective channels involved in the absorption and reabsorption of calcium across intestinal and kidney tubule epithelia (reviewed by [397, 428]).

**Table:** 

Nomenclature	TRPA1
HGNC, UniProt	*TRPA1*, O75762
Chemical activators	–
Other chemical activators	Isothiocyanates (covalent) and 1,4‐dihydropyridines (non‐covalent)
Physical activators	Cooling (<17^∘^C) (disputed)
Functional Characteristics	*γ* = 87‐100 pS; conducts mono‐ and di‐valent cations non‐selectively (P_*Ca*_/P_*Na*_ = 0.84); outward rectification; activated by elevated intracellular Ca^2+^
Activators	acrolein (Agonist) (pEC_50_ 5.3) [physiological voltage] [21], allicin (Agonist) (pEC_50_ 5.1) [physiological voltage] [22], *Δ*^9^‐tetrahydrocannabinol (Agonist) (pEC_50_ 4.9) [‐60mV] [158], nicotine (non‐covalent) (pEC_50_ 4.8) [‐75mV] [352], thymol (non‐covalent) (pEC_50_ 4.7) Concentration range: 6.2 × 10^−6^M‐2.5 × 10^−5^M [199], URB597 (Agonist) (pEC_50_ 4.6) [257], (‐)‐menthol (Partial agonist) (pEC_50_ 4–4.5) [164, 405], cinnamaldehyde (Agonist) (pEC_50_ 4.2) [physiological voltage] [15] – Mouse, icilin (Agonist) Concentration range: 1 × 10^−4^M [physiological voltage] [339] – Mouse
Selective activators	chlorobenzylidene malononitrile (covalent) (pEC_50_ 6.7) [37], formalin (covalent. This level of activity is also observed for rat TRPA1) (pEC_50_ 3.4) [228, 238] – Mouse
Channel blockers	AP18 (Inhibition) (pIC_50_ 5.5) [292], ruthenium red (Inhibition) (pIC_50_ 5.5) [‐80mV] [250] – Mouse, HC030031 (Inhibition) (pIC_50_ 5.2) [238]

**Table:** 

Nomenclature	TRPC1	TRPC2	TRPC3
HGNC, UniProt	*TRPC1*, P48995	*TRPC2*, –	*TRPC3*, Q13507
Chemical activators	NO‐mediated cysteine S‐nitrosation	–	diacylglycerols
Physical activators	membrane stretch (likely direct)	–
Functional Characteristics	It is not yet clear that TRPC1 forms a homomer. It does form heteromers with TRPC4 and TRPC5	–	*γ* = 66 pS; conducts mono and di‐valent cations non‐selectively (P_*Ca*_/P_*Na*_ = 1.6); monovalent cation current suppressed by extracellular Ca^2+^; dual (inward and outward) rectification
Activators	–	DOG (Agonist) Concentration range: 1 × 10^−4^M [‐80mV] [223] – Mouse, SAG (Agonist) Concentration range: 1 × 10^−4^M [‐80mV] [223] – Mouse	–
Channel blockers	2‐APB (Antagonist) [‐70mV] [342], Gd^3+^ (Antagonist) Concentration range: 2 × 10^−5^M [‐70mV] [427], GsMTx‐4, La^3+^ (Antagonist) Concentration range: 1 × 10^−4^M [‐70mV] [342], SKF96365	2‐APB (Antagonist) Concentration range: 5 × 10^−5^M [‐70mV – 80mV] [223] – Mouse	Gd^3+^ (Antagonist) (pEC_50_ 7) [‐60mV] [122], BTP2 (Antagonist) (pIC_50_ 6.5) [‐80mV] [126], La^3+^ (Antagonist) (pIC_50_ 5.4) [‐60mV] [122], 2‐APB (Antagonist) (pIC_50_ 5) [physiological voltage] [211], ACAA, KB‐R7943, Ni^2+^, Pyr3 [175], SKF96365

**Table:** 

Nomenclature	TRPC4	TRPC5	TRPC6	TRPC7
HGNC, UniProt	*TRPC4*, Q9UBN4	*TRPC5*, Q9UL62	*TRPC6*, Q9Y210	*TRPC7*, Q9HCX4
Chemical activators	–	–	–	diacylglycerols
Other chemical activators	NO‐mediated cysteine S‐nitrosation, potentiation by extracellular protons	NO‐mediated cysteine S‐nitrosation (disputed), potentiation by extracellular protons	Diacylglycerols	–
Physical activators	–	Membrane stretch (likely indirect)	Membrane stretch (likely indirect)	–
Functional Characteristics	*γ* = 30 ‐41 pS, conducts mono and di‐valent cations non‐selectively (P_*Ca*_/P_*Na*_ = 1.1 ‐ 7.7); dual (inward and outward) rectification	*γ* = 41‐63 pS; conducts mono‐and di‐valent cations non‐selectively (P_*Ca*_/P_*Na*_ = 1.8 ‐ 9.5); dual rectification (inward and outward) as a homomer, outwardly rectifying when expressed with TRPC1 or TRPC4	*γ* = 28‐37 pS; conducts mono and divalent cations with a preference for divalents (P_*Ca*_/P_*Na*_ = 4.5‐5.0); monovalent cation current suppressed by extracellular Ca^2+^ and Mg^2+^, dual rectification (inward and outward), or inward rectification	*γ* = 25‐75 pS; conducts mono and divalent cations with a preference for divalents (P_*Ca*_/ P_*Cs*_ = 5.9); modest outward rectification (monovalent cation current recorded in the absence of extracellular divalents); monovalent cation current suppressed by extracellular Ca^2+^ and Mg^2+^
Endogenous activators	–	intracellular Ca^2+^ (at negative potentials) (pEC_50_ 6.2), lysophosphatidylcholine	20‐HETE, arachidonic acid, lysophosphatidylcholine	–
Activators	La^3+^ (*μ*M range)	Gd^3+^ Concentration range: 1 × 10^−4^M, La^3+^ (*μ*M range), Pb^2+^ Concentration range: 5 × 10^−6^M, daidzein, genistein (independent of tyrosine kinase inhibition) [400]	flufenamate, hyp 9 [204], hyperforin [205]	–
Endogenous channel blockers	–	–	–	–
Channel blockers	ML204 (pIC_50_ 5.5) [240], 2‐APB, La^3+^ (mM range), SKF96365, niflumic acid (Antagonist) Concentration range: 3 × 10^−5^M [‐60mV] [380] – Mouse	KB‐R7943 (Inhibition) (pIC_50_ 5.9) [187], ML204 (pIC_50_∼5) [240], 2‐APB (Antagonist) (pIC_50_ 4.7) [‐80mV] [410], BTP2, GsMTx‐4, La^3+^ (Antagonist) Concentration range: 5 × 10^−3^M [‐60mV] [159] – Mouse, SKF96365, chlorpromazine, flufenamic acid	Gd^3+^ (Antagonist) (pIC_50_ 5.7) [‐60mV] [148] – Mouse, SKF96365 (Antagonist) (pIC_50_ 5.4) [‐60mV] [148] – Mouse, La^3+^ (pIC_50_∼5.2), amiloride (Antagonist) (pIC_50_ 3.9) [‐60mV] [148] – Mouse, Cd^2+^ (Antagonist) (pIC_50_ 3.6) [‐60mV] [148] – Mouse, 2‐APB, ACAA, GsMTx‐4, Extracellular H^+^, KB‐R7943, ML9	2‐APB, La^3+^ (Antagonist) Concentration range: 1 × 10^−4^M [‐60mV] [272] – Mouse, SKF96365 (Antagonist) Concentration range: 2.5 × 10^−5^M [‐60mV] [272] – Mouse, amiloride

**Table:** 

Nomenclature	TRPM1	TRPM2	TRPM3	TRPM4
HGNC, UniProt	*TRPM1*, Q7Z4N2	*TRPM2*, O94759	*TRPM3*, Q9HCF6	*TRPM4*, Q8TD43
Other channel blockers	–	–	–	Intracellular nucleotides including ATP, adenosine diphosphate, adenosine 5'‐monophosphate and AMP‐PNP with an IC_50_ range of 1.3‐1.9 *μ*M
Other chemical activators	–	Agents producing reactive oxygen (e.g. H_2_O_2_) and nitrogen (e.g. GEA 3162) species	–	–
Physical activators	–	Heat 35^∘^C	heat (Q_10_ = 7.2 between 15 ‐ 25^∘^C; Vriens *et al*., 2011), hypotonic cell swelling [376]	Membrane depolarization (V_1/2_ = ‐20 mV to + 60 mV dependent upon conditions) in the presence of elevated [Ca^2+^]_*i*_, heat (Q_10_ = 8.5 @ +25 mV between 15 and 25^∘^C)
Functional Characteristics	Conducts mono‐ and di‐valent cations non‐selectively, dual rectification (inward and outward)	*γ* = 52‐60 pS at negative potentials, 76 pS at positive potentials; conducts mono‐ and di‐valent cations non‐selectively (P_*Ca*_/P_*Na*_ = 0.6‐0.7); non‐rectifying; inactivation at negative potentials; activated by oxidative stress probably *via* PARP‐1, PARP inhibitors reduce activation by oxidative stress, activation inhibited by suppression of APDR formation by glycohydrolase inhibitors	TRPM3_1235_: *γ* = 83 pS (Na^+^ current), 65 pS (Ca^2+^ current); conducts mono and di‐valent cations non‐selectively (P_*Ca*_/P_*Na*_ = 1.6) TRPM3*α*1: selective for monovalent cations (P_*Ca*_/P_*Cs*_ 0.1); TRPM3*α*2: conducts mono‐ and di‐valent cations non‐selectively (P_*Ca*_/P_*Cs*_ = 1‐10); Outwardly rectifying (magnitude varies between spice variants)	*γ* = 23 pS (within the range 60 to +60 mV); permeable to monovalent cations; impermeable to Ca^2+^; strong outward rectification; slow activation at positive potentials, rapid deactivation at negative potentials, deactivation blocked by decavanadate
Endogenous activators	pregnenolone sulphate [194]	intracellular cADPR (Agonist) (pEC_50_ 5) [‐80mV – ‐60mV] [24, 184, 360], intracellular ADP ribose (Agonist) (pEC_50_ 3.9–4.4) [‐80mV] [289], intracellular Ca^2+^ (*via* calmodulin), H_2_O_2_ (Agonist) Concentration range: 5 × 10^−7^M‐5 × 10^−5^M [physiological voltage] [98, 123, 189, 332, 391], arachidonic acid (Potentiation) Concentration range: 1 × 10^−5^M‐3 × 10^−5^M [physiological voltage] [123]	sphingosine (Agonist) (pEC_50_ 4.9) [physiological voltage] [112], epipregnanolone sulphate [231], pregnenolone sulphate [377], sphinganine (Agonist) Concentration range: 2 × 10^−5^M [physiological voltage] [112]	intracellular Ca^2+^ (Agonist) (pEC_50_ 3.9–6.3) [‐100mV – 100mV] [259, 263, 264, 350]
Activators	–	GEA 3162	nifedipine	BTP2 (Agonist) (pEC_50_ 8.1) [‐80mV] [350], decavanadate (Agonist) (pEC_50_ 5.7) [‐100mV] [263]
Gating inhibitors	–	–	2‐APB (Antagonist) Concentration range: 1 × 10^−4^M [physiological voltage] [410]	flufenamic acid (Antagonist) (pIC_50_ 5.6) [100mV] [366] – Mouse, clotrimazole (Antagonist) Concentration range: 1 × 10^−6^M‐1 × 10^−5^M [100mV] [267]
Endogenous channel blockers	Zn^2+^ (pIC_50_ 6)	Zn^2+^ (pIC_50_ 6), extracellular H^+^	Mg^2+^ (Antagonist) Concentration range: 9 × 10^−3^M [‐80mV – 80mV] [269] – Mouse, extracellular Na^+^ (TRPM3*α*2 only)	–
Channel blockers		2‐APB (Antagonist) (pIC_50_ 6.1) [‐60mV] [361], ACAA (Antagonist) (pIC_50_ 5.8) [physiological voltage] [188], clotrimazole (Antagonist) Concentration range: 3 × 10^−6^M‐3 × 10^−5^M [‐60mV – ‐15mV] [131], econazole (Antagonist) Concentration range: 3 × 10^−6^M‐3 × 10^−5^M [‐60mV – ‐15mV] [131], flufenamic acid (Antagonist) Concentration range: 5 × 10^−5^M‐1 × 10^−3^M [‐60mV – ‐50mV] [130, 361], miconazole (Antagonist) Concentration range: 1 × 10^−5^M [‐60mV] [361]	Gd^3+^ (Antagonist) Concentration range: 1 × 10^−4^M [‐80mV – 80mV] [111, 198], La^3+^ (Antagonist) Concentration range: 1 × 10^−4^M [physiological voltage] [111, 198], mefenamic acid [177], pioglitazone (independent of PPAR‐*γ*) [232], rosiglitazone [232], troglitazone	9‐phenanthrol (pIC_50_ 4.6–4.8) [108], spermine (Antagonist) (pIC_50_ 4.2) [100mV] [265], adenosine (pIC_50_ 3.2)

**Table:** 

Nomenclature	TRPM5	TRPM6	TRPM7	TRPM8
HGNC, UniProt	*TRPM5*, Q9NZQ8	*TRPM6*, Q9BX84	*TRPM7*, Q96QT4	*TRPM8*, Q7Z2W7
EC number	–	2.7.11.1	2.7.11.1	–
Other chemical activators	–	constitutively active, activated by reduction of intracellular Mg^2+^	activation of PKA	agonist activities are temperature dependent and potentiated by cooling
Physical activators	membrane depolarization (V_1/2_ = 0 to + 120 mV dependent upon conditions), heat (Q_10_ = 10.3 @ ‐75 mV between 15 and 25^∘^C)	–	–	depolarization (V_1/2_ +50 mV at 15^∘^C), cooling (< 22‐26^∘^C)
Functional Characteristics	*γ* = 15‐25 pS; conducts monovalent cations selectively (P_*Ca*_/P_*Na*_ = 0.05); strong outward rectification; slow activation at positive potentials, rapid inactivation at negative potentials; activated and subsequently desensitized by [Ca^2+^]_*I*_	*γ*= 40‐87 pS; permeable to mono‐ and di‐valent cations with a preference for divalents (Mg^2+^> Ca^2+^; P_*Ca*_/P_*Na*_ = 6.9), conductance sequence Zn^2+^> Ba^2+^> Mg^2+^= Ca^2+^ = Mn^2+^> Sr^2+^> Cd^2+^> Ni^2+^; strong outward rectification abolished by removal of extracellular divalents, inhibited by intracellular Mg^2+^ (IC_50_ = 0.5 mM) and ATP	*γ* = 40‐105 pS at negative and positive potentials respectively; conducts mono‐and di‐valent cations with a preference for monovalents (P_*Ca*_/P_*Na*_ = 0.34); conductance sequence Ni^2+^> Zn^2+^> Ba^2+^ = Mg^2+^> Ca^2+^ = Mn^2+^> Sr^2+^> Cd^2+^; outward rectification, decreased by removal of extracellular divalent cations; inhibited by intracellular Mg^2+^, Ba^2+^, Sr^2+^, Zn^2+^, Mn^2+^ and Mg.ATP (disputed); activated by and intracellular alkalinization; sensitive to osmotic gradients	*γ* = 40‐83 pS at positive potentials; conducts mono‐ and di‐valent cations non‐selectively (P_*Ca*_/P_*Na*_ = 1.0‐3.3); pronounced outward rectification; demonstrates densensitization to chemical agonists and adaptation to a cold stimulus in the presence of Ca^2+^; modulated by lysophospholipids and PUFAs
Endogenous activators	intracellular Ca^2+^ (Agonist) (pEC_50_ 4.5–6.2) [‐80mV – 80mV] [139, 217, 366] – Mouse	extracellular H^+^ (Potentiation), intracellular Mg^2+^	intracellular ATP (Potentiation), Extracellular H^+^ (Potentiation), cyclic AMP (elevated cAMP levels)	–
Activators	–	2‐APB (Agonist) (pEC_50_ 3.4–3.7) [‐120mV – 100mV] [207]	2‐APB Concentration range: >1 × 10^−3^M [249] – Mouse	icilin (Agonist) (pEC_50_ 6.7–6.9) [physiological voltage] [10, 26] – Mouse, (‐)‐menthol (inhibited by intracellular Ca^2+^) (pEC_50_ 4.6) [‐120mV – 160mV] [371]
Selective activators	–	–	–	WS‐12 (Full agonist) (pEC_50_ 4.9) [physiological voltage] [224, 325] – Rat
Endogenous channel blockers	–	Mg^2+^ (inward current mediated by monovalent cations is blocked) (pIC_50_ 5.5–6), Ca^2+^ (inward current mediated by monovalent cations is blocked) (pIC_50_ 5.3–5.3)	–	–
Channel blockers	flufenamic acid (pIC_50_ 4.6), intracellular spermine (pIC_50_ 4.4), Extracellular H^+^ (pIC_50_ 3.2)	ruthenium red (pIC_50_ 7) [*voltage dependent* ‐120mV]	spermine (Inhibition) (p*K*_*i*_ 5.6) [‐110mV – 80mV] [186] – Rat, 2‐APB (Inhibition) (pIC_50_ 3.8) [‐100mV – 100mV] [207] – Mouse, carvacrol (Inhibition) (pIC_50_ 3.5) [‐100mV – 100mV] [276] – Mouse, Mg^2+^ (Antagonist) (pIC_50_ 2.5) [80mV] [249] – Mouse, La^3+^ (Antagonist) Concentration range: 2 × 10^−3^M [‐100mV – 100mV] [313] – Mouse	BCTC (Antagonist) (pIC_50_ 6.1) [physiological voltage] [26] – Mouse, 2‐APB (Antagonist) (pIC_50_ 4.9–5.1) [100mV – ‐100mV] [141, 254] – Mouse, capsazepine (Antagonist) (pIC_50_ 4.7) [physiological voltage] [26] – Mouse, *Δ*^9^‐tetrahydrocannabinol, 5‐benzyloxytryptamine, ACAA, AMTB [196], La^3+^, NADA, anandamide, cannabidiol, clotrimazole, linoleic acid
Comments	TRPM5 is not blocked by ATP	–	2‐APB acts as a channel blocker in the *μ*M range.	cannabidiol and *Δ*^9^‐tetrahydrocannabinol are examples of cannabinoids. TRPM8 is insensitive to ruthenium red. icilin requires intracellular Ca^2+^ for full agonist activity.

**Table:** 

Nomenclature	TRPML1	TRPML2	TRPML3
HGNC, UniProt	*MCOLN1*, Q9GZU1	*MCOLN2*, Q8IZK6	*MCOLN3*, Q8TDD5
Activators	TRPML1^*Va*^: Constitutively active, current potentiated by extracellular acidification (equivalent to intralysosomal acidification)	TRPML2^*Va*^: Constitutively active, current potentiated by extracellular acidification (equivalent to intralysosomal acidification)	TRPML3^*Va*^: Constitutively active, current inhibited by extracellular acidification (equivalent to intralysosomal acidicification) Wild type TRPML3: Activated by Na^+^‐free extracellular (extracytosolic) solution and membrane depolarization, current inhibited by extracellular acidification (equivalent to intralysosomal acidicification)
Functional Characteristics	TRPML1^*Va*^: *γ* = 40 pS and 76‐86 pS at very negative holding potentials with Fe^2+^ and monovalent cations as charge carriers, respectively; conducts Na^+^≈ K^+^>Cs^+^ and divalent cations (Ba^2+^>Mn2^+^>Fe^2+^>Ca^2+^> Mg^2+^> Ni^2+^>Co^2+^> Cd^2+^>Zn^2+^≫Cu^2+^) protons; monovalent cation flux suppressed by divalent cations (*e.g.* Ca^2+^, Fe^2+^); inwardly rectifying	TRPML1^*Va*^: Conducts Na^+^; monovalent cation flux suppressed by divalent cations; inwardly rectifying	TRPML3^*Va*^: *γ* = 49 pS at very negative holding potentials with monovalent cations as charge carrier; conducts Na^+^> K^+^> Cs^+^ with maintained current in the presence of Na^+^, conducts Ca^2+^ and Mg^2+^, but not Fe^2+^, impermeable to protons; inwardly rectifying Wild type TRPML3: *γ* = 59 pS at negative holding potentials with monovalent cations as charge carrier; conducts Na^+^> K^+^> Cs^+^ and Ca^2+^ (P_*Ca*_/P_*K*_≈ 350), slowly inactivates in the continued presence of Na^+^ within the extracellular (extracytosolic) solution; outwardly rectifying
Channel blockers	–	–	Gd^3+^ (Antagonist) (pIC_50_ 4.7) [‐80mV] [251] – Mouse

**Table:** 

Nomenclature	TRPP1	TRPP2	TRPP3
HGNC, UniProt	*PKD2*, Q13563	*PKD2L1*, Q9P0L9	*PKD2L2*, Q9NZM6
Activators	–	Calmidazolium (in primary cilia): 10 *μ*M	–
Functional Characteristics	The channel properties of TRPP1 (PKD2) have not been determined with certainty	Currents have been measured directly from primary cilia and also when expressed on plasma membranes. Primary cilia appear to contain heteromeric TRPP2 + PKD1‐L1, underlying a gently outwardly rectifying nonselective conductance (P_*Ca*_/P_*Na*_ 6: PKD1‐L1 is a 12 TM protein of unknown topology). Primary cilia heteromeric channels have an inward single channel conductance of 80 pS and an outward single channel conductance of 95 pS. Presumed homomeric TRPP2 channels are gently outwardly rectifying. Single channel conductance is 120 pS inward, 200 pS outward [74].	–
Channel blockers	–	phenamil (pIC_50_ 6.9), benzamil (pIC_50_ 6), ethylisopropylamiloride (pIC_50_ 5), amiloride (pIC_50_ 3.8), Gd^3+^ Concentration range: 1 × 10^−4^M [‐50mV] [54], La^3+^ Concentration range: 1 × 10^−4^M [‐50mV] [54], flufenamate	–

**Table:** 

Nomenclature	TRPV1	TRPV2	TRPV3
HGNC, UniProt	*TRPV1*, Q8NER1	*TRPV2*, Q9Y5S1	*TRPV3*, Q8NET8
Other chemical activators	NO‐mediated cysteine S‐nitrosation	–	NO‐mediated cysteine S‐nitrosytion
Physical activators	depolarization (V_1/2_ 0 mV at 35^∘^C), noxious heat (> 43^∘^C at pH 7.4)	noxious heat (> 35^∘^C; rodent, not human) [255]	depolarization (V_1/2_ +80 mV, reduced to more negative values following heat stimuli), heat (23^∘^C ‐ 39^∘^C, temperature threshold reduces with repeated heat challenge)
Functional Characteristics	*γ* = 35 pS at ‐ 60 mV; 77 pS at + 60 mV, conducts mono and divalent cations with a selectivity for divalents (P_*Ca*_/P_*Na*_ = 9.6); voltage‐ and time‐ dependent outward rectification; potentiated by ethanol; activated/potentiated/upregulated by PKC stimulation; extracellular acidification facilitates activation by PKC; desensitisation inhibited by PKA; inhibited by Ca^2+^/ calmodulin; cooling reduces vanilloid‐evoked currents; may be tonically active at body temperature	Conducts mono‐ and divalent cations (P_*Ca*_/P_*Na*_ = 0.9‐2.9); dual (inward and outward) rectification; current increases upon repetitive activation by heat; translocates to cell surface in response to IGF‐1 to induce a constitutively active conductance, translocates to the cell surface in response to membrane stretch	*γ* = 197 pS at = +40 to +80 mV, 48 pS at negative potentials; conducts mono‐ and divalent cations; outward rectification; potentiated by arachidonic acid
Endogenous activators	extracellular H^+^ (at 37^∘^C) (pEC_50_ 5.4), 12S‐HPETE (Agonist) (pEC_50_ 5.1) [‐60mV] [145] – Rat, 15S‐HPETE (Agonist) (pEC_50_ 5.1) [‐60mV] [145] – Rat, LTB_4_ (Agonist) (pEC_50_ 4.9) [‐60mV] [145] – Rat, 5S‐HETE	–	–
Activators	resiniferatoxin (Agonist) (pEC_50_ 8.4) [physiological voltage] [330], capsaicin (Agonist) (pEC_50_ 7.5) [‐100mV – 160mV] [371], camphor, diphenylboronic anhydride, phenylacetylrinvanil [13]	2‐APB (pEC_50_ 5) [255, 301] – Rat, *Δ*^9^‐tetrahydrocannabinol (pEC_50_ 4.8) [301] – Rat, cannabidiol (pEC_50_ 4.5) [301], probenecid (pEC_50_ 4.5) [16] – Rat, 2‐APB (Agonist) (pEC_50_ 3.8–3.9) [physiological voltage] [141, 160] – Mouse, diphenylboronic anhydride (Agonist) Concentration range: 1 × 10^−4^M [‐80mV] [61, 160] – Mouse	incensole acetate (pEC_50_ 4.8) [244] – Mouse, 2‐APB (Full agonist) (pEC_50_ 4.6) [‐80mV – 80mV] [62] – Mouse, diphenylboronic anhydride (Full agonist) (pEC_50_ 4.1–4.2) [*voltage dependent* ‐80mV – 80mV] [61] – Mouse, (‐)‐menthol (pEC_50_ 1.7) [‐80mV – 80mV] [227] – Mouse, camphor (Full agonist) Concentration range: 1 × 10^−3^M‐2 × 10^−3^M [‐60mV] [242] – Mouse, carvacrol (Full agonist) Concentration range: 5 × 10^−4^M [‐80mV – 80mV] [408] – Mouse, eugenol (Full agonist) Concentration range: 3 × 10^−3^M [‐80mV – 80mV] [408] – Mouse, thymol (Full agonist) Concentration range: 5 × 10^−4^M [‐80mV – 80mV] [408] – Mouse
Selective activators	olvanil (Agonist) (pEC_50_ 7.7) [physiological voltage] [330], DkTx (pEC_50_ 6.6) [physiological voltage] [33] – Rat	–	6‐tert‐butyl‐*m*‐cresol (pEC_50_ 3.4) [374] – Mouse
Channel blockers	5'‐iodoresiniferatoxin (pIC_50_ 8.4), 6‐iodo‐nordihydrocapsaicin (pIC_50_ 8), BCTC (Antagonist) (pIC_50_ 7.5) [52], capsazepine (Antagonist) (pIC_50_ 7.4) [‐60mV] [237], ruthenium red (pIC_50_ 6.7–7), 2‐APB, NADA, allicin, anandamide	ruthenium red (pIC_50_ 6.2), La^3+^, SKF96365, TRIM (Inhibition) Concentration range: 5 × 10^−4^M [160] – Mouse, amiloride	diphenyltetrahydrofuran (Antagonist) (pIC_50_ 5–5.2) [‐80mV – 80mV] [61] – Mouse, ruthenium red (Inhibition) Concentration range: 1 × 10^−6^M [‐60mV] [286] – Mouse
Selective channel blockers	AMG517 (pIC_50_ 9) [31], AMG628 (pIC_50_ 8.4) [383] – Rat, A425619 (pIC_50_ 8.3) [91], A778317 (pIC_50_ 8.3) [28], SB366791 (pIC_50_ 8.2) [119], JYL1421 (Antagonist) (pIC_50_ 8) [388] – Rat, JNJ17203212 (Antagonist) (pIC_50_ 7.8) [physiological voltage] [345], SB705498 (Antagonist) (pIC_50_ 7.1) [118], SB452533	–	–
Labelled ligands	[^3^H]A778317 (Channel blocker) (p*K*_*d*_ 8.5) [28], [^125^I]resiniferatoxin (Channel blocker, Antagonist) (pIC_50_ 8.4) [‐50mV] [378] – Rat, [^3^H]resiniferatoxin (Activator)	–	–

**Table:** 

Nomenclature	TRPV4	TRPV5	TRPV6
HGNC, UniProt	*TRPV4*, Q9HBA0	*TRPV5*, Q9NQA5	*TRPV6*, Q9H1D0
Activators	–	constitutively active (with strong buffering of intracellular Ca^2+^)	constitutively active (with strong buffering of intracellular Ca^2+^)
Other channel blockers	–	Pb^2+^ = Cu^2+^ = Gd^3+^>Cd^2+^>Zn^2+^>La^3+^>Co^2+^> Fe^2^	–
Other chemical activators	Epoxyeicosatrieonic acids and NO‐mediated cysteine S‐nitrosylation	–	–
Physical activators	Constitutively active, heat (> 24^∘^C ‐ 32^∘^C), mechanical stimuli	–	–
Functional Characteristics	*γ* = 60 pS at ‐60 mV, 90‐100 pS at +60 mV; conducts mono‐ and di‐valent cations with a preference for divalents (P_*Ca*_/P_*Na*_ =6‐10); dual (inward and outward) rectification; potentiated by intracellular Ca^2+^ *via* Ca^2+^/ calmodulin; inhibited by elevated intracellular Ca^2+^ *via* an unknown mechanism (IC_50_ = 0.4 *μ*M)	*γ* = 59‐78 pS for monovalent ions at negative potentials, conducts mono‐ and di‐valents with high selectivity for divalents (P_*Ca*_/P_*Na*_> 107); voltage‐ and time‐ dependent inward rectification; inhibited by intracellular Ca^2+^ promoting fast inactivation and slow downregulation; feedback inhibition by Ca^2+^ reduced by calcium binding protein 80‐K‐H; inhibited by extracellular and intracellular acidosis; upregulated by 1,25‐dihydrovitamin D3	*γ* = 58‐79 pS for monovalent ions at negative potentials, conducts mono‐ and di‐valents with high selectivity for divalents (P_*Ca*_/P_*Na*_> 130); voltage‐ and time‐dependent inward rectification; inhibited by intracellular Ca^2+^ promoting fast and slow inactivation; gated by voltage‐dependent channel blockade by intracellular Mg^2+^; slow inactivation due to Ca^2+^‐dependent calmodulin binding; phosphorylation by PKC inhibits Ca^2+^‐calmodulin binding and slow inactivation; upregulated by 1,25‐dihydroxyvitamin D3
Activators	phorbol 12‐myristate 13‐acetate (Agonist) (pEC_50_ 7.9) [physiological voltage] [406]	–	2‐APB (Potentiation)
Selective activators	GSK1016790A (pEC_50_ 8.7) [physiological voltage] [359], 4*α*‐PDH (pEC_50_ 7.1) [physiological voltage] [176] – Mouse, RN1747 (pEC_50_ 6.1) [physiological voltage] [370], bisandrographolide (Agonist) (pEC_50_ 6) [‐60mV] [333] – Mouse, 4*α*‐PDD (Agonist) Concentration range: 3 × 10^−7^M [physiological voltage] [406]	–	–
Channel blockers	Gd^3+^, La^3+^, ruthenium red (Inhibition) Concentration range: 1 × 10^−6^M [physiological voltage] [154], ruthenium red (Inhibition) Concentration range: 2 × 10^−7^M [physiological voltage] [116] – Rat	ruthenium red (pIC_50_ 6.9), Mg^2+^, econazole, miconazole	ruthenium red (Antagonist) (pIC_50_ 5) [‐80mV] [136] – Mouse, Cd^2+^, La^3+^, Mg^2+^
Selective channel blockers	HC067047 (Inhibition) (pIC_50_ 7.3) [‐40mV] [93], RN1734 (Inhibition) (pIC_50_ 5.6) [physiological voltage] [370]	–	–

### Comments

### TRPA (ankyrin) family

Agents activating TRPA1 in a covalent manner are thiol reactive electrophiles that bind to cysteine and lysine residues within the cytoplasmic domain of the channel [133, 225]. TRPA1 is activated by a wide range of endogenous and exogenous compounds and only a few representative examples are mentioned in the table: an exhaustive listing can be found in [17]. In addition, TRPA1 is potently activated by intracellular zinc (EC_50_= 8 nM) [11, 140].

### TRPM (melastatin) family

Ca^2+^ activates all splice variants of TRPM2, but other activators listed are effective only at the full length isoform [87]. Inhibition of TRPM2 by clotrimazole, miconazole, econazole, flufenamic acid is largely irreversible. TRPM4 exists as multiple spice variants: data listed are for TRPM4b. The sensitivity of TRPM4b and TRPM5 to activation by [Ca^2+^]_*i*_ demonstrates a pronounced and time‐dependent reduction following excision of inside‐out membrane patches [366]. The V_1/2_ for activation of TRPM4 and TRPM5 demonstrates a pronounced negative shift with increasing temperature. Activation of TRPM8 by depolarization is strongly temperature‐dependent via a channel‐closing rate that decreases with decreasing temperature. The V_1/2_ is shifted in the hyperpolarizing direction both by decreasing temperature and by exogenous agonists, such as (‐)‐menthol[371] whereas antagonists produce depolarizing shifts in V_1/2_[247]. The V_1/2_ for the native channel is far more positive than that of heterologously expressed TRPM8 [247]. It should be noted that (‐)‐menthol and structurally related compounds can elicit release of Ca^2+^ from the endoplasmic reticulum independent of activation of TRPM8 [229]. Intracellular pH modulates activation of TRPM8 by cold and icilin, but not (‐)‐menthol[10].

### TRPML (mucolipin) family

Data in the table are for TRPML proteins mutated (*i.e* TRPML1^*Va*^, TRPML2^*Va*^ and TRPML3^*Va*^) at loci equivalent to TRPML3 A419P to allow plasma membrane expression when expressed in HEK‐293 cells and subsequent characterisation by patch‐clamp recording [85, 109, 169, 251, 409]. Data for wild type TRPML3 are also tabulated [169, 170, 251, 409]. It should be noted that alternative methodologies, particularly in the case of TRPML1, have resulted in channels with differing biophysical characteristics (reviewed by [297]).

### TRPP (polycystin) family

Data in the table are extracted from [72, 80] and [326]. Broadly similar single channel conductance, mono‐ and di‐valent cation selectivity and sensitivity to blockers are observed for TRPP2 co‐expressed with TRPP1 [79]. Ca^2+^, Ba^2+^ and Sr^2+^ permeate TRPP3, but reduce inward currents carried by Na^+^. Mg^2+^ is largely impermeant and exerts a voltage dependent inhibition that increases with hyperpolarization.

### TRPV (vanilloid) family

Activation of TRPV1 by depolarisation is strongly temperature‐dependent via a channel opening rate that increases with increasing temperature. The V_1/2_ is shifted in the hyperpolarizing direction both by increasing temperature and by exogenous agonists [371]. The sensitivity of TRPV4 to heat, but not 4*α*‐PDD is lost upon patch excision. TRPV4 is activated by anandamide and arachidonic acid following P450 epoxygenase‐dependent metabolism to 5,6‐epoxyeicosatrienoic acid (reviewed by [266]). Activation of TRPV4 by cell swelling, but not heat, or phorbol esters, is mediated via the formation of epoxyeicosatrieonic acids. Phorbol esters bind directly to TRPV4. TRPV5 preferentially conducts Ca^2+^ under physiological conditions, but in the absence of extracellular Ca^2+^, conducts monovalent cations. Single channel conductances listed for TRPV5 and TRPV6 were determined in divalent cation‐free extracellular solution. Ca^2+^‐induced inactivation occurs at hyperpolarized potentials when Ca^2+^ is present extracellularly. Single channel events cannot be resolved (probably due to greatly reduced conductance) in the presence of extracellular divalent cations. Measurements of P_*Ca*_/P_*Na*_ for TRPV5 and TRPV6 are dependent upon ionic conditions due to anomalous mole fraction behaviour. Blockade of TRPV5 and TRPV6 by extracellular Mg^2+^ is voltage‐dependent. Intracellular Mg^2+^ also exerts a voltage dependent block that is alleviated by hyperpolarization and contributes to the time‐dependent activation and deactivation of TRPV6 mediated monovalent cation currents. TRPV5 and TRPV6 differ in their kinetics of Ca^2+^‐dependent inactivation and recovery from inactivation. TRPV5 and TRPV6 function as homo‐ and hetero‐tetramers.

### Further Reading

Baraldi PG *et al*. (2010) Transient receptor potential ankyrin 1 (TRPA1) channel as emerging target for novel analgesics and anti‐inflammatory agents. *J. Med. Chem.* **53**: 5085‐107 [PMID:20356305]

Cheng KT *et al*. (2011) Contribution of TRPC1 and Orai1 to Ca(2+) entry activated by store depletion. *Adv. Exp. Med. Biol.* **704**: 435‐49 [PMID:21290310]

Clapham DE *et al*. (2003) International Union of Pharmacology. XLIII. Compendium of voltage‐gated ion channels: transient receptor potential channels. *Pharmacol. Rev.* **55**: 591‐6 [PMID:14657417]

Everaerts W *et al*. (2010) The vanilloid transient receptor potential channel TRPV4: from structure to disease. *Prog. Biophys. Mol. Biol.* **103**: 2‐17 [PMID:19835908]

Guinamard R *et al*. (2011) The non‐selective monovalent cationic channels TRPM4 and TRPM5. *Adv. Exp. Med. Biol.* **704**: 147‐71 [PMID:21290294]

Gunthorpe MJ *et al*. (2009) Clinical development of TRPV1 antagonists: targeting a pivotal point in the pain pathway. *Drug Discov. Today* **14**: 56‐67 [PMID:19063991]

Harteneck C *et al*. (2011) Pharmacological modulation of diacylglycerol‐sensitive TRPC3/6/7 channels. *Curr Pharm Biotechnol* **12**: 35‐41 [PMID:20932261]

Harteneck C *et al*. (2011) Synthetic modulators of TRP channel activity. *Adv. Exp. Med. Biol.* **704**: 87‐106 [PMID:21290290]

Islam MS. (2011) TRP channels of islets. *Adv. Exp. Med. Biol.* **704**: 811‐30 [PMID:21290328]

Knowlton WM *et al*. (2011) TRPM8: from cold to cancer, peppermint to pain. *Curr Pharm Biotechnol* **12**: 68‐77 [PMID:20932257]

Koike C *et al*. (2010) TRPM1: a vertebrate TRP channel responsible for retinal ON bipolar function. *Cell Calcium* **48**: 95‐101 [PMID:20846719]

Liu Y *et al*. (2011) TRPM8 in health and disease: cold sensing and beyond. *Adv. Exp. Med. Biol.* **704**: 185‐208 [PMID:21290296]

Mälkiä A *et al*. (2011) The emerging pharmacology of TRPM8 channels: hidden therapeutic potential underneath a cold surface. *Curr Pharm Biotechnol* **12**: 54‐67 [PMID:20932258]

Nilius B *et al*. (2010) Transient receptor potential channelopathies. *Pflugers Arch.* **460**: 437‐50 [PMID:20127491]

Nilius B *et al*. (2007) Transient receptor potential cation channels in disease. *Physiol. Rev.* **87**: 165‐217 [PMID:17237345]

Owsianik G *et al*. (2006) Permeation and selectivity of TRP channels. *Annu. Rev. Physiol.* **68**: 685‐717 [PMID:16460288]

Ramsey IS *et al*. (2006) An introduction to TRP channels. *Annu. Rev. Physiol.* **68**: 619‐47 [PMID:16460286]

Rohacs T. (2009) Phosphoinositide regulation of non‐canonical transient receptor potential channels. *Cell Calcium* **45**: 554‐65 [PMID:19376575]

Runnels LW. (2011) TRPM6 and TRPM7: A Mul‐TRP‐PLIK‐cation of channel functions. *Curr Pharm Biotechnol* **12**: 42‐53 [PMID:20932259]

Vay L *et al*. (2011) The thermo‐TRP ion channel family: properties and therapeutic implications. *Br J Pharmacol* [PMID:21797839]

Vennekens R *et al*. (2008) Vanilloid transient receptor potential cation channels: an overview. *Curr. Pharm. Des.* **14**: 18‐31 [PMID:18220815]

Vincent F *et al*. (2011) TRPV4 agonists and antagonists. *Curr Top Med Chem* **11**: 2216‐26 [PMID:21671873]

Vriens J *et al*. (2009) Pharmacology of vanilloid transient receptor potential cation channels. *Mol. Pharmacol.* **75**: 1262‐79 [PMID:19297520]

Wu LJ *et al*. (2010) International Union of Basic and Clinical Pharmacology. LXXVI. Current progress in the mammalian TRP ion channel family. *Pharmacol. Rev.* **62**: 381‐404 [PMID:20716668]

Yamamoto S *et al*. (2010) Chemical physiology of oxidative stress‐activated TRPM2 and TRPC5 channels. *Prog. Biophys. Mol. Biol.* **103**: 18‐27 [PMID:20553742]

Yuan JP *et al*. (2009) TRPC channels as STIM1‐regulated SOCs. *Channels (Austin)* **3**: 221‐5 [PMID:19574740]

Zeevi DA *et al*. (2007) TRPML and lysosomal function. *Biochim. Biophys. Acta* **1772**: 851‐8 [PMID:17306511]

Zholos A. (2010) Pharmacology of transient receptor potential melastatin channels in the vasculature. *Br. J. Pharmacol.* **159**: 1559‐71 [PMID:20233227]

## Voltage‐gated calcium channels

### Overview

Calcium (Ca^2+^) channels are voltage‐gated ion channels present in the membrane of most excitable cells. The nomenclature for Ca^2+^channels was proposed by [92] and **approved by the NC‐IUPHAR Subcommittee on Ca^2+^ channels** [**50**]. Ca^2+^ channels form hetero‐oligomeric complexes. The *α*1 subunit is pore‐forming and provides the binding site(s) for practically all agonists and antagonists. The 10 cloned *α*1‐subunits can be grouped into three families: (1) the high‐voltage activated dihydropyridine‐sensitive (L‐type, Ca_*V*_1.x) channels; (2) the high‐voltage activated dihydropyridine‐insensitive (Ca_*V*_2.x) channels and (3) the low‐voltage‐activated (T‐type, Ca_*V*_3.x) channels. Each *α*1 subunit has four homologous repeats (I‐IV), each repeat having six transmembrane domains and a pore‐forming region between transmembrane domains S5 and S6. Gating is thought to be associated with the membrane‐spanning S4 segment, which contains highly conserved positive charges. Many of the *α*1‐subunit genes give rise to alternatively spliced products. At least for high‐voltage activated channels, it is likely that native channels comprise co‐assemblies of *α*1, *β* and *α*2‐*δ* subunits. The *γ* subunits have not been proven to associate with channels other than the *α*1s skeletal muscle Cav1.1 channel. The *α*2‐*δ*1 and *α*2‐*δ*2 subunits bind gabapentin and pregabalin.

**Table:** 

Nomenclature	Ca_*v*_1.1	Ca_*v*_1.2	Ca_*v*_1.3	Ca_*v*_1.4	Ca_*v*_2.1
HGNC, UniProt	*CACNA1S*, Q13698	*CACNA1C*, Q13936	*CACNA1D*, Q01668	*CACNA1F*, O60840	*CACNA1A*, O00555
Functional Characteristics	L‐type calcium current: High voltage‐activated, slow voltage dependent inactivation	L‐type calcium current: High voltage‐activated, slow voltage‐dependent inactivation, rapid calcium‐dependent inactivation	L‐type calcium current: Voltage‐activated, slow voltage‐dependent inactivation, more rapid calcium‐dependent inactivation	L‐type calcium current: Moderate voltage‐activated, slow voltage‐dependent inactivation	P/Q‐type calcium current: Moderate voltage‐activated, moderate voltage‐dependent inactivation
Activators	(‐)‐(S)‐BayK8644, FPL64176, SZ(+)‐(*S*)‐202‐791	(‐)‐(S)‐BayK8644, FPL64176 Concentration range: 1 × 10^−6^M‐5 × 10^−6^M [219] – Rat, SZ(+)‐(*S*)‐202‐791	(‐)‐(S)‐BayK8644	(‐)‐(S)‐BayK8644	–
Gating inhibitors	nifedipine (Antagonist)	nifedipine (Antagonist)	nitrendipine (Inhibition) (pIC_50_ 8.4) [329]	–	–
Selective gating inhibitors	–	–	–	–	*ω*‐agatoxin IVA (P current component: Kd = 2nM, Q component Kd= >100nM) (pIC_50_ 7–8.7) [‐100mV – ‐90mV] [34, 241] – Rat, *ω*‐agatoxin IVB (p*K*_*d*_ 8.5) [‐80mV] [4] – Rat
Channel blockers	diltiazem (Antagonist), verapamil (Antagonist)	diltiazem (Antagonist), verapamil (Antagonist)	verapamil (Antagonist)	–	–
(Sub)family‐selective channel blockers	calciseptine (Antagonist)	calciseptine (Antagonist)	–	–	*ω*‐conotoxin MVIIC (pIC_50_ 8.2–9.2) Concentration range: 2 × 10^−6^M‐5 × 10^−6^M [physiological voltage] [206] – Rat
Comments	–	–	Ca_*v*_1.3 activates more negative potentials than Ca_*v*_1.2 and is incompletely inhibited by dihydropyridine antagonists.	Ca_*v*_1.4 is less sensitive to dihydropyridine antagonists than other Cav1 channels	–

**Table:** 

Nomenclature	Ca_*v*_2.2	Ca_*v*_2.3	Ca_*v*_3.1	Ca_*v*_3.2	Ca_*v*_3.3
HGNC, UniProt	*CACNA1B*, Q00975	*CACNA1E*, Q15878	*CACNA1G*, O43497	*CACNA1H*, O95180	*CACNA1I*, Q9P0X4
Functional Characteristics	N‐type calcium current: High voltage‐activated, moderate voltage‐dependent inactivation	R‐type calcium current: Moderate voltage‐activated, fast voltage‐dependent inactivation	T‐type calcium current: Low voltage‐activated, fast voltage‐dependent inactivation	T‐type calcium current: Low voltage‐activated, fast voltage‐dependent inactivation	T‐type calcium current: Low voltage‐activated, moderate voltage‐dependent inactivation
Gating inhibitors	–	–	kurtoxin (Antagonist) (pIC_50_ 7.3–7.8) [‐90mV] [58, 327] – Rat	kurtoxin (Antagonist) (pIC_50_ 7.3–7.6) [‐90mV] [58, 327] – Rat	–
Selective gating inhibitors	–	SNX482 (Antagonist) (pIC_50_ 7.5–8) [physiological voltage] [256]	–	–	–
Channel blockers	–	Ni^2+^ (Antagonist) (pIC_50_ 4.6) [‐90mV] [396]	mibefradil (Antagonist) (pIC_50_ 6–6.6) [‐110mV – ‐100mV] [234], Ni^2+^ (Antagonist) (pIC_50_ 3.6–3.8) [*voltage dependent* ‐90mV] [197] – Rat	mibefradil (Antagonist) (pIC_50_ 5.9–7.2, median 6.8) [‐110mV – ‐80mV] [234], Ni^2+^ (Antagonist) (pIC_50_ 4.9–5.2) [*voltage dependent* ‐90mV] [197]	mibefradil (Antagonist) (pIC_50_ 5.8) [‐110mV] [234], Ni^2+^ (Antagonist) (pIC_50_ 3.7–4.1) [*voltage dependent* ‐90mV] [197] – Rat
(Sub)family‐selective channel blockers	*ω*‐conotoxin GVIA (Antagonist) (pIC_50_ 10.4) [‐80mV] [206] – Rat, *ω*‐conotoxin MVIIC (Antagonist) (pIC_50_ 6.1–8.5, median 8.2) [‐80mV] [132, 206, 236] – Rat	–	–	–	–

### Comments

In many cell types, P and Q current components cannot be adequately separated and many researchers in the field have adopted the terminology ‘P/Q‐type’ current when referring to either component. Both of these physiologically defined current types are conducted by alternative forms of Cav2.1. Ziconotide (a synthetic peptide equivalent to *ω*‐conotoxin MVIIA) has been approved for the treatment of chronic pain [395].

## Voltage‐gated proton channel

### Overview

The voltage‐gated proton channel (provisionally denoted H_*v*_1) is a putative 4TM proton‐selective channel gated by membrane depolarization and which is sensitive to the transmembrane pH gradient [45, 75, 76, 305, 317]. The structure of H_*v*_1 is homologous to the voltage sensing domain (VSD) of the superfamily of voltage‐gated ion channels (*i.e*. segments S1 to S4) and contains no discernable pore region [305, 317]. Proton flux through H_*v*_1 is instead most likely mediated by a water wire completed in a crevice of the protein when the voltage‐sensing S4 helix moves in response to a change in transmembrane potential [304, 401]. H_*v*_1 expresses largely as a dimer mediated by intracellular C‐terminal coiled‐coil interactions [208] but individual promoters nonetheless support gated H^+^ flux via separate conduction pathways [182, 200, 291, 362]. Within dimeric structures, the two protomers do not function independently, but display co‐operative interactions during gating resulting in increased voltage sensitivity, but slower activation, of the dimeric,*versus* monomeric, complexes [107, 363] .

**Table:** 

Nomenclature	H_*v*_1
HGNC, UniProt	*HVCN1*, Q96D96
Functional Characteristics	Activated by membrane depolarization mediating macroscopic currents with time‐, voltage‐ and pH‐dependence; outwardly rectifying; voltage dependent kinetics with relatively slow current activation sensitive to extracellular pH and temperature, relatively fast deactivation; voltage threshold for current activation determined by pH gradient (*Δ*pH = pH_*o*_ ‐pH_*i*_) across the membrane
Channel blockers	Zn^2+^ (pIC_50_∼5.7–6.3), Cd^2+^ (pIC_50_∼5)

### Comments

The voltage threshold (V_*thr*_) for activation of Hv1 is not fixed but is set by the pH gradient across the membrane such that V_*thr*_ is positive to the Nernst potential for H^+^, which ensures that only outwardly directed flux of H^+^ occurs under physiological conditions [45, 75, 76]. Phosphorylation of H_*v*_1 within the N‐terminal domain by PKC enhances the gating of the channel [245]. Tabulated IC_50_ values for Zn^2^+ and Cd^2+^ are for heterologously expressed human and mouse H_*v*_1 [305, 317]. Zn^2+^ is not a conventional pore blocker, but is coordinated by two, or more, external protonation sites involving histamine residues [305]. Zn^2+^ binding may occur at the dimer interface between pairs of histamine residues from both monomers where it may interfere with channel opening [246]. Mouse knockout studies demonstrate that H_*v*_1 participates in charge compensation in granulocytes during the respiratory burst of NADPH oxidase‐dependent reactive oxygen species production that assists in the clearance of bacterial pathogens [306]. Additional physiological functions of H_*v*_1 are reviewed by [45].

## Voltage‐gated sodium channels

### Overview

Sodium channels are voltage‐gated sodium‐selective ion channels present in the membrane of most excitable cells. Sodium channels comprise of one pore‐forming *α* subunit, which may be associated with either one or two *β* subunits [152]. *α*‐Subunits consist of four homologous domains (I‐IV), each containing six transmembrane segments (S1‐S6) and a pore‐forming loop. The positively charged fourth transmembrane segment (S4) acts as a voltage sensor and is involved in channel gating. The crystal structure of the bacterial NavAb channel has revealed a number of novel structural features compared to earlier potassium channel structures including a short selectivity filter with ion selectivity determined by interactions with glutamate side chains [280]. Interestingly, the pore region is penetrated by fatty acyl chains that extend into the central cavity which may allow the entry of small, hydrophobic pore‐blocking drugs [280]. Auxiliary *β*1, *β*2, *β*3 and *β*4 subunits consist of a large extracellular N‐terminal domain, a single transmembrane segment and a shorter cytoplasmic domain.

**The nomenclature for sodium channels was proposed by Goldin *et al*., (2000)** [**105**] **and approved by the NC‐IUPHAR Subcommittee on sodium channels (Catterall *et al*., 2005,** [**48**]).

**Table:** 

Nomenclature	Na_*v*_1.1	Na_*v*_1.2	Na_*v*_1.3	Na_*v*_1.4	Na_*v*_1.5
HGNC, UniProt	*SCN1A*, P35498	*SCN2A*, Q99250	*SCN3A*, Q9NY46	*SCN4A*, P35499	*SCN5A*, Q14524
Functional Characteristics	Activation V_0.5_ = ‐20 mV. Fast inactivation (*τ* = 0.7 ms for peak sodium current).	Activation V_0.5_ = ‐24 mV. Fast inactivation (*τ* = 0.8 ms for peak sodium current).	Activation V_0.5_ = ‐24 mV. Fast inactivation (0.8 ms)	Activation V_0.5_ = ‐30 mV. Fast inactivation (0.6 ms)	Activation V_0.5_ = ‐26 mV. Fast inactivation (*τ* = 1 ms for peak sodium current).
(Sub)family‐selective activators	batrachotoxin, veratridine	batrachotoxin (Agonist) (p*K*_*d*_ 9.1) [physiological voltage] [213] – Rat, veratridine (Partial agonist) (p*K*_*d*_ 5.2) [physiological voltage] [49] – Rat	batrachotoxin, veratridine	batrachotoxin (Full agonist) Concentration range: 5 × 10^−6^M [‐100mV] [386] – Rat, veratridine (Partial agonist) Concentration range: 2 × 10^−4^M [‐100mV] [386] – Rat	batrachotoxin (Full agonist) (p*K*_*d*_ 7.6) [physiological voltage] [324] – Rat, veratridine (Partial agonist) (pEC_50_ 6.3) [‐30mV] [381] – Rat
(Sub)family‐selective channel blockers	saxitoxin (Pore blocker), tetrodotoxin (Pore blocker) Concentration range: 1 × 10^−8^M	saxitoxin (Pore blocker) (pIC_50_ 8.8) [‐120mV] [36] – Rat, tetrodotoxin (Pore blocker) (pIC_50_ 8) [‐120mV] [36] – Rat, lacosamide (Antagonist) (pIC_50_ 4.5) [‐80mV] [1] – Rat	tetrodotoxin (Pore blocker) (pIC_50_ 8.4) [55], saxitoxin (Pore blocker)	saxitoxin (Pore blocker) (pIC_50_ 8.4) [‐100mV] [288] – Rat, tetrodotoxin (Pore blocker) (pIC_50_ 7.6) [‐120mV] [51], *μ*‐conotoxin GIIIA (Pore blocker) (pIC_50_ 5.9) [‐100mV] [51]	tetrodotoxin (Pore blocker) (p*K*_*d*_ 5.8) [‐80mV] [69, 418] – Rat

**Table:** 

Nomenclature	Na_*v*_1.6	Na_*v*_1.7	Na_*v*_1.8	Na_*v*_1.9
HGNC, UniProt	*SCN8A*, Q9UQD0	*SCN9A*, Q15858	*SCN10A*, Q9Y5Y9	*SCN11A*, Q9UI33
Functional Characteristics	Activation V_0.5_ = ‐29 mV. Fast inactivation (1 ms)	Activation V_0.5_ = ‐27 mV. Fast inactivation (0.5 ms)	Activation V_0.5_ = ‐16 mV. Inactivation (6 ms)	Activation V_0.5_ = ‐32 mV. Slow inactivation (16 ms)
(Sub)family‐selective activators	batrachotoxin, veratridine	batrachotoxin, veratridine	–	–
(Sub)family‐selective channel blockers	tetrodotoxin (Pore blocker) (pIC_50_ 9) [‐130mV] [83] – Rat, saxitoxin (Pore blocker)	tetrodotoxin (Pore blocker) (pIC_50_ 7.6) [‐100mV] [178], saxitoxin (Pore blocker) (pIC_50_ 6.2) [379]	tetrodotoxin (Pore blocker) (pIC_50_ 4.2) [‐60mV] [5] – Rat	tetrodotoxin (Pore blocker) (pIC_50_ 4.4) [‐120mV] [70] – Rat
Selective channel blockers	–	–	PF‐01247324 (Pore blocker) (pIC_50_ 6.7) [*voltage dependent*] [281]	–

### Comments

Sodium channels are also blocked by local anaesthetic agents, antiarrythmic drugs and antiepileptic drugs. In general, these drugs are not highly selective among channel subtypes. There are two clear functional fingerprints for distinguishing different subtypes. These are sensitivity to tetrodotoxin(Na_*V*_1.5, Na_*V*_1.8 and Na_*V*_1.9 are much less sensitive to block) and rate of fast inactivation (Na_*V*_1.8 and particularly Na_*V*_1.9 inactivate more slowly). All sodium channels also have a slow inactivation process that is engaged during long depolarizations (>100 msec) or repetitive trains of stimuli. All sodium channel subtypes are blocked by intracellular QX‐314.
